# New mechanistic understanding of osteoclast differentiation and bone resorption mediated by P2X7 receptors and PI3K-Akt-GSK3β signaling

**DOI:** 10.1186/s11658-024-00614-5

**Published:** 2024-07-08

**Authors:** Jiajia Lu, Xiaojian Shi, Qiang Fu, Yaguang Han, Lei Zhu, Zhibin Zhou, Yongchuan Li, Nan Lu

**Affiliations:** 1 Department of Orthopedic Trauma, Shanghai Fourth People’s Hospital, School of Medicine, Tongji University, 1279 Sanmen Road, Shanghai, 200434 China; 2 Department of Orthopedic Trauma, Shanghai Changzheng Hospital, Shanghai, 200434 China; 3 Department of Orthopedic Trauma, Haimen People’s Hospital of Jiangsu Province, Nantong, 226100 China; 4 Department of Orthopaedics, General Hospital of Northern Theater Command, No. 83, Culture Road, Shenhe District, Shenyang, 110016 Liaoning China

**Keywords:** Osteoporosis, Osteoclasts, P2X7 receptors, PI3K-Akt-GSK3β signaling pathway, Transcriptomics, Metabolomics

## Abstract

**Objective:**

Osteoporosis is a global health issue characterized by decreased bone mass and microstructural degradation, leading to an increased risk of fractures. This study aims to explore the molecular mechanism by which P2X7 receptors influence osteoclast formation and bone resorption through the PI3K-Akt-GSK3β signaling pathway.

**Methods:**

An osteoporosis mouse model was generated through ovariectomy (OVX) in normal C57BL/6 and P2X7^f/f^; LysM-cre mice. Osteoclasts were isolated for transcriptomic analysis, and differentially expressed genes were selected for functional enrichment analysis. Metabolite analysis was performed using liquid chromatography-tandem mass spectrometry (LC–MS/MS), and multivariate statistical analysis and pattern recognition were used to identify differential lipid metabolism markers and their distribution. Bioinformatics analyses were conducted using the Encyclopedia of Genes and Genomes database and the MetaboAnalyst database to assess potential biomarkers and create a metabolic pathway map. Osteoclast precursor cells were used for in vitro cell experiments, evaluating cell viability and proliferation using the Cell Counting Kit 8 (CCK-8) assay. Osteoclast precursor cells were induced to differentiate into osteoclasts using macrophage colony-stimulating factor (M-CSF) and receptor activator of nuclear factor kappa-beta ligand (RANKL), and tartrate-resistant acid phosphatase (TRAP) staining was performed to compare differentiation morphology, size, and quantity between different groups. Western blot analysis was used to assess the expression of differentiation markers, fusion gene markers, and bone resorption ability markers in osteoclasts. Immunofluorescence staining was employed to examine the spatial distribution and quantity of osteoclast cell skeletons, P2X7 protein, and cell nuclei, while pit assay was used to evaluate osteoclast bone resorption ability. Finally, in vivo animal experiments, including micro computed tomography (micro-CT), hematoxylin and eosin (HE) staining, TRAP staining, and immunohistochemistry, were conducted to observe bone tissue morphology, osteoclast differentiation, and the phosphorylation level of the PI3K-Akt-GSK3β signaling pathway.

**Results:**

Transcriptomic and metabolomic data collectively reveal that the P2X7 receptor can impact the pathogenesis of osteoporosis through the PI3K-Akt-GSK3β signaling pathway. Subsequent in vitro experiments showed that cells in the Sh-P2X7 + Recilisib group exhibited increased proliferative activity (1.15 versus 0.59), higher absorbance levels (0.68 versus 0.34), and a significant increase in resorption pit area (13.94 versus 3.50). Expression levels of osteoclast differentiation-related proteins MMP-9, CK, and NFATc1 were markedly elevated (MMP-9: 1.72 versus 0.96; CK: 2.54 versus 0.95; NFATc1: 3.05 versus 0.95), along with increased fluorescent intensity of F-actin rings. In contrast, the OE-P2X7 + LY294002 group showed decreased proliferative activity (0.64 versus 1.29), reduced absorbance (0.34 versus 0.82), and a significant decrease in resorption pit area (5.01 versus 14.96), accompanied by weakened expression of MMP-9, CK, and NFATc1 (MMP-9: 1.14 versus 1.79; CK: 1.26 versus 2.75; NFATc1: 1.17 versus 2.90) and decreased F-actin fluorescent intensity. Furthermore, in vivo animal experiments demonstrated that compared with the wild type (WT) + Sham group, mice in the WT + OVX group exhibited significantly increased levels of CTX and NTX in serum (CTX: 587.17 versus 129.33; NTX: 386.00 versus 98.83), a notable decrease in calcium deposition (19.67 versus 53.83), significant reduction in bone density, increased trabecular separation, and lowered bone mineral density (BMD). When compared with the KO + OVX group, mice in the KO + OVX + recilisib group showed a substantial increase in CTX and NTX levels in serum (CTX: 503.50 versus 209.83; NTX: 339.83 versus 127.00), further reduction in calcium deposition (29.67 versus 45.33), as well as decreased bone density, increased trabecular separation, and reduced BMD.

**Conclusion:**

P2X7 receptors positively regulate osteoclast formation and bone resorption by activating the PI3K-Akt-GSK3β signaling pathway.

**Supplementary Information:**

The online version contains supplementary material available at 10.1186/s11658-024-00614-5.

## Introduction

Osteoporosis (OP) is a common chronic metabolic bone disease characterized by bone loss and degradation of bone microstructure, leading to a significant increase in the risk of fractures [1–3]. This condition profoundly affects the quality of life and health status of the elderly population [4, 5]. Globally, the incidence of osteoporosis is as high as 18% [6], and with the exacerbation of the aging trend in the population, its incidence is also on the rise, posing a global health issue [7, 8]. However, current osteoporosis treatments, such as bisphosphonates and hormonal drugs, exhibit significant adverse reactions [9]. Therefore, gaining an in-depth understanding of the pathogenesis of OP and identifying new therapeutic targets and strategies are of utmost importance [10, 11].

The main features of OP are reduced bone density and deteriorated bone microstructure, resulting in skeletal weakening and susceptibility to fractures [12–14]. Osteoclasts play a critical role in the development of OP, and their differentiation and activity are crucial for the regulation of bone resorption [15, 16]. Under normal circumstances, bone resorption and bone formation are in a balanced state, maintaining skeletal stability [17, 18]. However, in osteoporosis, there is enhanced activity of osteoclasts, leading to increased bone resorption relative to bone formation, resulting in decreased bone density and degradation of bone microstructure [19, 20].

Recent studies have shown that the ion channel receptor P2X7 receptor plays a crucial role in the development of osteoporosis [21, 22]. P2X7 receptor is highly expressed in osteoclasts and is involved in regulating cell differentiation and bone resorption processes [23, 24]. Research has demonstrated that P2X7 gene knockout mice exhibit increased bone mass deposition [25], highlighting its close interplay with osteoporosis. P2X7 functions through mediating multiple signaling pathways in bone diseases [26, 27], with the PI3K-Akt-GSK3β signaling pathway being a crucial pathway in osteoporosis-related research. The activation of the PI3K-AKT-GSK3β pathway is closely linked to the activation of caspase-3 and apoptosis-related signals, which are also implicated in osteoclast apoptosis in bone disease studies [28]. Furthermore, activation of this pathway can enhance osteoclast differentiation and bone resorption capacity [29, 30]. However, the precise mechanisms of action of the P2X7 receptor and the PI3K-Akt-GSK3β signaling pathway in osteoporosis are not yet fully understood [31, 32].

The scientific and clinical significance of this research lies in further investigating the regulatory mechanisms of the P2X7 receptor and the PI3K-Akt-GSK3β signaling pathway in OP. By gaining an in-depth understanding of the role of this signaling pathway in osteoclast differentiation and bone resorption, new targets and strategies can be identified for the treatment and prevention of OP. Furthermore, this research holds important clinical implications for understanding the molecular mechanisms underlying the occurrence and progression of OP, as well as exploring novel therapeutic approaches. With the results of this study, we aim to provide new breakthroughs for early diagnosis and treatment of OP, contributing to the improvement of patients’ quality of life.

## Materials and methods

### Ethical statement

This study strictly adheres to international guidelines and principles for animal experimentation to ensure that experiments are carried out with minimal animal suffering. Prior to the start of the experiments, we obtained approval from the animal ethics committee at Shanghai Fourth People’s Hospital, School of Medicine, Tongji University, on 27 February 2023 (approval number: TJBH12523101). All experimental animals were housed in specific pathogen-free (SPF) environments, provided with adequate food and water, and given appropriate resting conditions. Humane methods were employed for animal disposal after the experiments were concluded. And the animal ethics committee at Shanghai Fourth People’s Hospital, School of Medicine, Tongji University follows the rules of Basel Declaration.

### Experimental animals

To obtain P2X7^f/f^; LysM-cre mice, we performed crosses between P2X7-flox/flox (NM-CKO-220373, Nanmo Biotech) mice and LysM-cre (NM-KI-215037, Nanmo Biotech) mice using the Cre-loxP system. All mice were bred under SPF conditions and fed with specialized feed and water provided by Beijing Weitonglihua Experimental Technology Company. Additionally, temperature and lighting conditions were strictly controlled to ensure consistency throughout the experiment.

### Establishment of the OP model

Ovariectomy (OVX) was performed to induce osteoporosis in female C57BL/6 mice aged 8–10 weeks and P2X7f/f; LysM-cre mice. The procedure involved the following steps: mice were anesthetized with pentobarbital sodium (328,510-1G, Merck) at a dose of 50 mg/kg. A small incision was made in the abdominal cavity of the mice to locate and expose the ovaries. Using surgical forceps, the fallopian tubes, ovaries, and surrounding tissues were separated to ensure complete ovary removal, followed by wound closure. The success rate of the osteoporosis model was determined by calculating the ratio of mice with successful modeling to the total number of mice operated on. At week 8, micro computed tomography (micro-CT) was employed to evaluate bone density, fracture rate, and other bone parameters in the mice, comparing them with the control group to assess the success of the model establishment. Throughout the experiment, 90% of the mice were retained in the successful modeling group for further research.

In the first part, the mice were divided into two groups, each comprising three mice: (1) wild type (WT) + OVX group, in which OVX was performed on C57BL/6 mice; (2) KO + OVX group, where OVX was conducted on P2X7f/f; LysM-cre mice.

In the second part, as depicted in Fig. S1, the modeling process entailed categorizing the mice into four groups, with each group consisting of six mice: (1) WT + Sham group, involving sham surgery on WT mice; specifically, they were anesthetized akin to the OVX procedure, a simulated incision was made without affecting ovarian tissues, followed by disinfection of the surgical site. Sampling occurred at week 8; (2) WT + OVX group, where WT mice underwent OVX and were sampled at week 8; (3) KO + OVX group, where P2X7f/f; LysM-cre mice underwent OVX and were sampled at week 8; (4) KO + OVX + Recilisib group, where P2X7f/f; LysM-cre mice underwent OVX and initiated daily intraperitoneal injections of recilisib (HY-101625, MCE) the day after surgery, with sampling at week 8.

For Recilisib, the concentration administered was 10 mg/kg, with an injection volume of 100 μL. The formulation was prepared according to the following ratio: 10% dimethylsulfoxide (DMSO), 40% polyethylene glycol 300 (PEG300; HY-Y0873, MCE), 5% Tween 80 (HY-Y1891, MCE), and 45% saline [33].

### RNA extraction and transcriptome sequencing

For the partial transcriptome sequencing of animals, we selected the femur tissue of mice, with three mice per group. The tissue (20–50 mg) was cut on a dry ice platform for sequencing. Total RNA was extracted from the tissue using TRIzol (catalog number: 15596026, ThermoFisher, USA), and the purity and concentration of the extracted RNA were assessed using a nanodrop2000 spectrophotometer (ThermoFisher, USA). Following the instructions of the PrimeScript RT reagent Kit (catalog number: RR047A, Takara, Japan), the RNA was reverse transcribed into cDNA for transcriptome sequencing. Differential analysis was performed using the “limma” package in R, with a cutoff of |log2(FoldChange)|> 2 and a significance threshold of *P* < 0.05 for selecting differentially expressed genes [34].

### LC–MS/MS analysis of metabolites

Mouse fecal samples were collected from the established C57BL/6 and P2X7^f/f^; LysM-cre mice OP model, with ten samples per group. All samples were stored at −80 °C to maintain stability until further analysis. After collecting fecal samples from each cage, they were freeze-dried and ground using a centrifugal grinding mill (Retsch, Haan, Germany). The samples were then added to precooled 50% methanol and thoroughly mixed by vortexing. After incubating on ice for 5 min, the supernatant was collected by centrifugation at 4 °C and 15,000 *g* for 15 min for subsequent analysis. Metabolite analysis was performed using liquid chromatography-tandem mass spectrometry (LC–MS/MS). Protein precipitation and metabolite extraction of the samples were carried out using methanol (HPLC grade, Merck, Germany). The samples were chromatographically separated using a Vanquish UHPLC system (100 × 2.1 mm, 1.9 μm) at a constant temperature of 40 °C, and the eluted metabolites were detected using an Orbitrap Q Exactive series mass spectrometer (Thermo Fisher). A C18 column was used in the UHPLC-MS/MS analysis. The sample injection volume was 5 mL, and the column flow rate was maintained at 0.2 mL/min. The mobile phase consisted of two eluents. In positive mode, eluent A was 0.1% formic acid (FA) in water, and eluent B was methanol. In negative mode, eluent A was a 5 mM ammonium acetate solution at pH 9.0, and eluent B was methanol. The gradient elution was as follows: 2% B for 1.5 min, 2–100% B for 12.0 min, 100% B for 14.0 min, 100–2% B for 14.1 min, and finally 2% B for 17 min. The spray voltage of the mass spectrometer was set at 3.2 kV, the capillary temperature was 320 °C, the gas flow rate was set at 35 arb, and the auxiliary gas flow rate was set at 10 arb.

The collected data were initially processed using MassHunter Workstation Software (Agilent, USA) to extract the mass spectrometric features of the metabolites. Subsequently, SIMCA software (Umetrics, Sweden) was used for multivariate statistical analysis, including principal component analysis (PCA) and orthogonal projections to latent structure discriminant analysis (OPLS-DA), to identify differential lipid metabolite markers and their distribution [35].

The correlation between differential metabolites and differential genes was analyzed using the Pearson correlation method, and the correlation relationship was visualized using the “ggplot” package in R to generate a heatmap.

### Functional enrichment analysis

The core gene set and metabolites were subjected to pathway analysis using the Kyoto Encyclopedia of Genes and Genomes (KEGG) and Reactome databases. Firstly, the core gene set and metabolites obtained from transcriptomics and metabolomics data were uploaded to the database’s analysis tools. We specifically focused on information related to the PI3K-Akt-GSK3β signaling pathway to better understand its role and importance in the study [35]. All the analyses were performed using R language and Bioconductor packages.

### Isolation of osteoclast precursor cells from C57BL/6 mouse

Bone marrow macrophages (BMM) were extracted from C57BL/6 mice for subsequent investigation of osteoclast differentiation and absorption. After ethical handling, mouse femurs were extracted, and bone marrow cells were separated from osteoclast precursor cells by density gradient centrifugation. The bone marrow was placed in a culture dish containing trypsin to release the bone marrow cells, and the bone marrow was digested and separated using the enzyme solution in the culture dish to release the osteoclast precursor cells. The bone marrow cells were purified and separated through multiple centrifugation steps. In the first centrifugation, cells were centrifuged at 300 *g* for 5 min to remove most cell debris and tissue residues, and the cell-containing supernatant was collected. The supernatant was transferred to a new centrifuge tube, and larger cells were precipitated by increasing the centrifugation speed and time to 800 *g* for 10 min. Finally, centrifugation at 1200 *g* for 15 min was performed to precipitate and separate the BMM cells. The cells were cultured in Roswell Park Memorial Institute (RPMI)-1640 medium containing 10% fetal bovine serum (16,140,089, Gibco, USA) and 1% antibiotic mixture (15,140,122, Invitrogen, USA) for subsequent experiments [36].

Flow cytometry was used to identify the isolated primary BMM cells. The isolated cells were collected and washed twice with phosphate buffered saline (PBS) containing 1% fetal bovine serum, each time at 300 *g* for 5 min. Then, 1 × 10^6^ cells were suspended in a 96-well plate with 200 μl per well. The cells were labeled with diluted antibodies in the dark at 4 °C for 30 min. The antibodies used were f4/80-PE (ab237335, Abcam) and CD11b-FITC (ab24874, Abcam). After labeling, the cells were washed twice with PBS containing 1% fetal bovine serum to remove excess antibodies. Finally, cell analysis was performed using the FACS Calibur flow cytometer (BD Biosciences, USA). FlowJo software was used for data analysis to calculate the proportion of cells labeled as f4/80^+^ CD11b^+^.

### Constructing lentiviruses for gene silencing and overexpression

The lentivirus packaging service was provided by GenBio Co., Ltd. (Shanghai, China). pHAGE-puro plasmids and auxiliary plasmids pSPAX2, pMD2.G, and pSuper-retro-puro plasmids and auxiliary plasmids gag/pol, VSVG were cotransfected into 293T cells (CRL-3216, ATCC, USA). After 48 h of cell culture, the supernatant was collected, and the filtered supernatant, after centrifugation through a 0.45 μm filter, was collected as the virus. After 72 h, the supernatant was harvested again and centrifuged for concentration. The two rounds of virus were mixed, and the titer was determined. The sequences of the siRNAs used for lentiviral silencing are shown in Table S1 and were validated for silencing efficiency in bone marrow-derived mesenchymal stem cells (BMSCs) cell lines (PCS-500–012, ATCC, USA).

As shown in Fig. S2, the sequence (Sh-P2X7-2) with the most optimal silencing effect was selected for subsequent experiments.

### Cell treatment and grouping

In vitro experiments were performed using bone marrow-derived osteoclast precursors (BMM) obtained from C57BL/6 mice. For lentivirus-mediated cell transfection, 5 × 10^5^ cells were seeded into 6-well plates. When the confluence of BMSCs reached 70–90%, the culture medium containing an appropriate amount of packaged lentivirus [multiplicity of infection (MOI) = 10, working titer approximately 5 × 10^6^ TU/mL] and 5 μg/mL polybrene (TR-1003, Merck, USA) was added for transfection. After 4 h of transfection, an equal amount of culture medium was added to dilute polybrene. The medium was replaced with a fresh culture medium after 24 h of transfection, and the transfection was observed by the luciferase reporter gene after 48 h. G418 (A1113803, Gibco, Grand Island, NY, USA) was used for resistance selection to obtain stable cell lines. Cells were collected when they no longer died in the presence of G418, and silencing efficiency was confirmed by reverse transcription-quantitative polymerase chain reaction (RT-qPCR) [37].

In the first part, cells were divided into four groups: (1) Sh-NC group: transfected with lentivirus carrying empty vector for silencing; (2) Sh-P2X7 group: transfected with lentivirus carrying P2X7 vector for silencing; (3) OE-NC group: transfected with lentivirus carrying empty vector for overexpression; and (4) OE-P2X7 group: transfected with lentivirus carrying P2X7 vector for overexpression. The working titer of lentivirus for all groups was 5 × 10^6^ TU/mL.

In the second part, cells were divided into four groups: (1) Sh-P2X7 + DMSO group: transfected with lentivirus carrying P2X7 vector for silencing, treated with 10 μL DMSO; (2) Sh-P2X7 + recilisib group: transfected with lentivirus carrying P2X7 vector for silencing, treated with 10 μM recilisib (HY-101625, MCE); (3) OE-P2X7 + DMSO group: transfected with lentivirus carrying P2X7 vector for overexpression, treated with 10 μL DMSO; (4) OE-P2X7 + LY294002 group: transfected with lentivirus carrying P2X7 vector for overexpression, treated with 10 μM LY294002 (HY-10108, MCE).

After the respective treatments, all cells were induced for osteoclast differentiation using M-CSF (50 ng/mL) and RANKL (50 ng/mL; 462-TEC-010, R&D, USA). Cells were cultured in the differentiation medium for 7 days for subsequent experiments [38].

### Cell viability and proliferation assay

Cell viability and proliferation were assessed using the Cell Counting Kit 8 (CCK-8) assay kit (CK04, Dojindo, Japan). After pretreating the cells according to the aforementioned grouping method, a single-cell suspension was prepared by diluting it in a complete culture medium to a concentration of 5 × 10^4^ cells/mL. Subsequently, 100 μL of the cell suspension was seeded into each well of a 96-well plate. The outer wells of the plate were filled with PBS solution, and the plate was then incubated at 37 °C with 5% CO_2_ for 24 h. The supernatant was discarded, and the plate was washed twice with PBS. Subsequently, six wells were randomly assigned for each group and incubated for an additional 24 and 48 h, respectively. To each well, 10 μL of CCK-8 solution was added, mixed well, and incubated at 37 °C with 5% CO_2_. Finally, the absorbance at 450 nm was measured [39].

### Tartrate-resistant acid phosphatase (TRAP) staining

The processed cells were fixed with 10% formaldehyde at room temperature for 10 min. Subsequently, they were stained using the TRAP acid phosphatase assay kit (P0332, Beyotime) following the manufacturer’s protocol. After staining, the cells were transferred to a new culture dish and treated with an equal volume of TRAP solution [containing 4.93 mg p-nitrophenyl phosphate (PNPP) in 0.5 M acetic acid solution (750 ml) and 150 ml tartaric acid solution] for 30 min at 37 °C. The detection buffer from the kit served as the negative control. The reaction was stopped by adding 0.5 M sodium hydroxide (NaOH), and the absorbance was measured at a wavelength of 405 nm using a Multiskan™ FC microplate reader (1,410,101, ThermoFisher).

For tissues, paraffin-embedded sections were deparaffinized in a gradient of ethanol to expose the tissue. After deparaffinization, the tissue sections were incubated in TRAP staining solution at 37 °C for 30 min, followed by washing off the excess staining solution with PBS. The sections were then mounted, observed, and captured under a microscope. Three slices were selected from each animal, and three fields were randomly chosen from each slice for image capture. The number of TRAP-positive cells was counted [40].

### Bone resorption pit detection

The bone resorption assay kit (BRA-24KIT, Cosmo Bio, Japan) was used. Following the grouping of BMM cells as described above, they were added to the wells of the kit’s plate along with the assay buffer. Detection was carried out at excitation and emission wavelengths of 485 nm and 535 nm, respectively. Subsequently, cells were removed after treatment with 5% sodium hypochlorite, washed with PBS, and observed under a microscope for the assessment of their resorption activity. The area of resorption pits was measured, calculated, and subjected to statistical analysis [40].

### F-actin staining

To assess the formation of F-actin rings, osteoclasts were first fixed in a 4% paraformaldehyde solution at room temperature for 15 min and then washed with PBS. After treatment with 0.3% Triton X-100, the osteoclasts were stained with rhodamine-labeled phalloidin (ab176753, Abcam) at room temperature for 60 min, followed by staining with 4′,6-diamidino-2-phenylindole (DAPI; 10,236,276,001, Sigma-Aldrich, USA) for 5 min at room temperature. Formation of F-actin rings was observed using a fluorescence microscope (IX71, Olympus, Japan) and statistically analyzed using ImageJ software [41].

### Micro-CT analysis

In this study, we utilized the mCT-40 micro-CT system from Scanco Medical (Switzerland) to scan the region of interest in the femoral bone tissue and analyze its growth. The scanning parameters employed were as follows: power current (385 μA), power voltage (65 kV), pixel size (9 μm), filter (AI 1.0 mm), and rotation step (0.4°). The reconstructed images were generated using Bruker’s NRecon software, and the data were analyzed using the CTAn program. Specifically, we determined the volume of interest (VOI) at 0.5 mm above and 0.25 mm in height from the growth plate of the femoral head. Within this volume, the region of interest (ROI) was manually defined as the subchondral area of cartilage, with a constant threshold (50–255) applied for trabecular bone binary segmentation. The micro-CT parameters assessed included: (a) bone volume fraction (BV/TV), which represents the ratio of bone surface area to tissue volume; (b) trabecular separation (Tb/Sp), indicating the distance between trabeculae within the bone; (c) trabecular thickness (Tb.Th), reflecting the average thickness of trabeculae and describing their structural changes; (d) trabecular number (Tb.N), which counts the intersections between bone and nonbone tissue within a given length; (e) bone mineral density (BMD), a measure of bone quantity and distribution density [42, 43].

### Hematoxylin and Eosin (HE) staining

After sample collection, decalcification was performed followed by paraffin embedding. Histological examination of tissue morphology was conducted using 4 μm thick sections stained with hematoxylin and eosin (HE). Paraffin sections were deparaffinized in xylene and different concentrations of ethanol (100%, 95%, 80%, 70%), followed by immersion in tap water for 15 min. The sections were then stained with hematoxylin staining solution (H8070, Solarbio, Beijing, China) for 5–10 min at room temperature. Subsequently, the slides were rinsed with distilled water, dehydrated in 95% ethanol, and placed in eosin staining solution (G1100, Solarbio, Beijing, China) for 5–10 min at room temperature, followed by standard dehydration, clearing, and mounting procedures [44].

### Von Kossa staining

Mouse femoral tissue sections were stained using the calcium salt staining kit (G3282, Solarbio) for Von Kossa staining. The sections were immersed in 1% silver nitrate and exposed to bright light for 30 min, followed by three washes with deionized water. Subsequently, 5% sodium thiosulfate was added and incubated for 5 min to remove unreacted silver. Calcium phosphate deposits were visualized in black (room temperature). The stained sections were observed and captured under an optical microscope [45]. The Von Kossa control solution from the kit was used as the negative control reagent.

### Immunohistochemical staining

Mouse femoral tissue sections were dehydrated in 100% ethanol, 95% ethanol, and 70% ethanol, followed by rinsing with water. Antigen retrieval was conducted in a high-temperature, high-pressure environment. The activity of endogenous peroxidases was reduced using a 3% hydrogen peroxide solution. Subsequently, cover slips were incubated overnight at 4 °C with specified primary antibodies: rabbit anti-p-PI3K (ab182651, 1:1000, Abcam), rabbit anti-p-AKT (ab38449, 1:1000), and rabbit anti-p-GSK3β (ab68476, 1:1000, Abcam). PBS solution was used as the negative control reagent. Following three washes with PBS, 100 μL of enzyme-labeled goat anti-rabbit immunoglobulin G (IgG) polymer (pv6000, Zhongshanjinqiao, China) was added and incubated for 30 min. Subsequent color development was carried out using the DAB chromogenic kit (ZLI-9018, Zhongshanjinqiao, China) at room temperature, and the staining was observed under a microscope. Images were captured post-staining. In total, there were six animals per group, with three sections stained per animal and three fields selected for imaging. After imaging, the positive area percentage was calculated using ImageJ 1.48u software (V1.48, National Institutes of Health, USA) [46].

### Enzyme-linked immunosorbent assay (ELISA)

We used a mouse Cross-linked Type I Collagen C-Telopeptide (CTX) ELISA kit (M3023, Elabscience, Wuhan, China) and a mouse Cross-linked Type I Collagen N-Telopeptide (NTX) ELISA kit (E-EL-M3022, Elabscience, Wuhan, China) for mice. Following the instructions, we analyzed the levels of bone turnover markers CTX and NTX in the serum of the model mice. Firstly, standards and test samples were added to each well and incubated at 37 °C for 90 min. After that, the liquid was removed, and 100 μL of biotinylated antibody working solution was added and incubated at 37 °C for another 60 min. After three washes, 100 μL of horseradish peroxidase (HRP) conjugate working solution was added and incubated at 37 °C for 30 min. The liquid was removed, and the wells were washed five times. Then, 90 μL of substrate solution was added to each well and incubated for approximately 15 min at 37 °C. Finally, 50 μL of stop solution was added to each well, and the absorbance (OD value) was measured at 450 nm wavelength using a microplate reader to calculate the sample concentration. The blank well was set as zero [40].

### Western blot

The mouse femoral tissue and cells were ground and homogenized in radioimmunoprecipitation assay (RIPA) lysis buffer (P0013B, Beyotime Biotechnology, China) and a protease inhibitor (P1005, Bi Yun Tian, China). Digestion of the cells was performed on ice. After grinding, the mixture was left to stand at 4 °C for 1 h, followed by centrifugation at 12,000 rpm for 15 min at 4 °C to extract the supernatant, which was then stored at −80 °C. The protein concentration was determined using a BCA assay kit (A53226, Thermo Fisher Scientific, Rockford, IL, USA). The proteins were transferred from the polyacrylamide gel to a polyvinylidene fluoride (PVDF) membrane (IPVH85R, Millipore, Darmstadt, Germany) using a wet transfer method. The membrane was then blocked with 5% bovine serum albumin (BSA) at room temperature for 1 h, followed by an incubation with the following primary antibodies: rabbit anti-P2X7 (ab307718, 1 µg/ml, Abcam), rabbit anti-MMP9 (ab283575, 1 µg/ml, Abcam), rabbit anti-CK (ab53280, 0.1 µg/ml, Abcam), rabbit anti-NFATc1 (A303-508A-T, 1 µg/ml, ThermoFisher), rabbit anti-PI3K (ab302958, 1 µg/ml, Abcam), rabbit anti-p-PI3K (ab182651, 2 µg/ml, Abcam), rabbit anti-AKT (ab8805, 1 µg/ml), rabbit anti-p-AKT (ab38449, 1 µg/ml), rabbit anti-GSK3β (ab185141, 1 µg/ml), rabbit anti-p-GSK3β (ab68476, 1 µg/ml, Abcam), mouse anti-β-actin (3700, 1 µg/ml, Cell Signaling Technology, USA), rabbit anti-Tub (ab131034, 1 µg/ml, Abcam), and mouse anti-glyceraldehyde 3-phosphate dehydrogenase (GAPDH; ab8245, 1 µg/ml, Abcam). After washing, the membrane was incubated with an HRP-conjugated anti-rabbit IgG secondary antibody (ab6721, 0.2 µg/ml, Abcam) or an HRP-conjugated anti-mouse IgG secondary antibody (ab205719, 0.2 µg/ml, Abcam) for 1 h. Detection was carried out using a chemiluminescence imager. Protein quantification analysis was performed with ImageJ 1.48u software, calculating the ratio of grayscale values of each protein to a reference grayscale value [47]. The experiment was repeated three times.

### RT-qPCR

Total RNA from the cells was extracted using TRIzol (catalog number: 15596026, ThermoFisher, USA). The purity and concentration of the extracted RNA were assessed using a nanodrop2000 spectrophotometer (ThermoFisher, USA). The RNA was reverse transcribed into cDNA using the PrimeScript RT reagent Kit (RR047A, Takara, Japan), according to the manufacturer’s instructions. RT-qPCR was performed using the Fast SYBR Green PCR kit (11,736,059, Thermo Fisher Scientific, China) with three replicates per well, using GAPDH as an internal reference. The relative expression level was calculated using the 2^−ΔΔCt^ method. The experiment was repeated three times. The primer sequences used in this study are provided in Table S2 [48].

### Statistical analysis

All data were analyzed using GraphPad Prism 8.0. Continuous data are presented as mean ± standard deviation (Mean ± SD). Unpaired *t*-tests were used to compare data between two groups, while a one-way analysis of variance (ANOVA) was used to compare multiple groups.

The homogeneity of variances was tested using Levene’s test, and if the variances were homogenous, Dunnett’s *t*-test and LSD-*t* test were used for pairwise comparisons. If the variances were not homogenous, Dunnett’s T3 test was used. Pearson’s analysis was used to evaluate the correlation between genes and immune cell content. *P* < 0.05 was considered statistically significant for the differences between the two groups [49].

## Results

### P2X7 receptor influences OP via the PI3K-AKT signaling pathway

Osteoporosis (OP) is a bone metabolism disorder primarily characterized by decreased bone mass and microstructural deterioration, largely caused by increased osteoclast activity. The P2X7 receptor plays a crucial role in bone diseases. To further investigate the specific role of P2X7 in the pathogenesis of OP, we utilized the Cre-loxP system to generate P2X7^f/f^; LysM-cre mice by crossing P2X7^f/f^ and LysM-cre mice. Through western blot and RT-qPCR analyses of the femoral tissues of the mice, we assessed the expression levels of P2X7. The results revealed that in the knockout (KO) group of mice, both protein and mRNA levels of P2X7 were no longer expressed (Fig. S3A, B), confirming the successful establishment of P2X7^f/f^; LysM-cre mice. Subsequently, we established an osteoporosis (OP) model by performing ovariectomy (OVX) on WT C57BL/6 and KO-P2X7 P2X7f/f; LysM-cre mice, with Sham groups set as controls. The expression levels of P2X7 in mouse bone tissues were assessed using western blot and RT-qPCR. We observed a significant increase in P2X7 expression in the femoral tissues of WT + OVX mice compared with WT + Sham mice (relative protein expression: 1.06 versus 1.83; relative mRNA expression: 0.97 versus 2.58). Conversely, mice in both KO + Sham and KO + OVX groups continued to show no expression of P2X7 (Fig. S3C, D). Subsequently, through HE staining, we observed that femoral tissues of mice in the WT + Sham group and KO + Sham group exhibited normal bone tissue morphology, with well-organized and dense trabeculae, and no fatty deposits in the bone marrow cavity. In contrast, femoral tissues of mice in the WT + OVX group displayed sparse and disordered trabeculae, large gaps, and visible fat tissue filling the bone marrow cavity, showing typical osteoporotic morphological features. The KO + OVX group showed some improvement compared with the WT + OVX group, with reduced gaps between trabeculae (Figure S3E). Additionally, micro CT imaging and its statistical analysis revealed that the WT + Sham and KO + Sham mice had a higher number of trabeculae with a certain thickness in their femoral tissues. The WT + OVX mice showed fewer trabeculae, lower thickness, increased dispersion, and significant pathological changes, such as a narrow growth plate. The KO + OVX group displayed relatively milder characteristics (Fig. S3F, G).

**Fig. 1:**
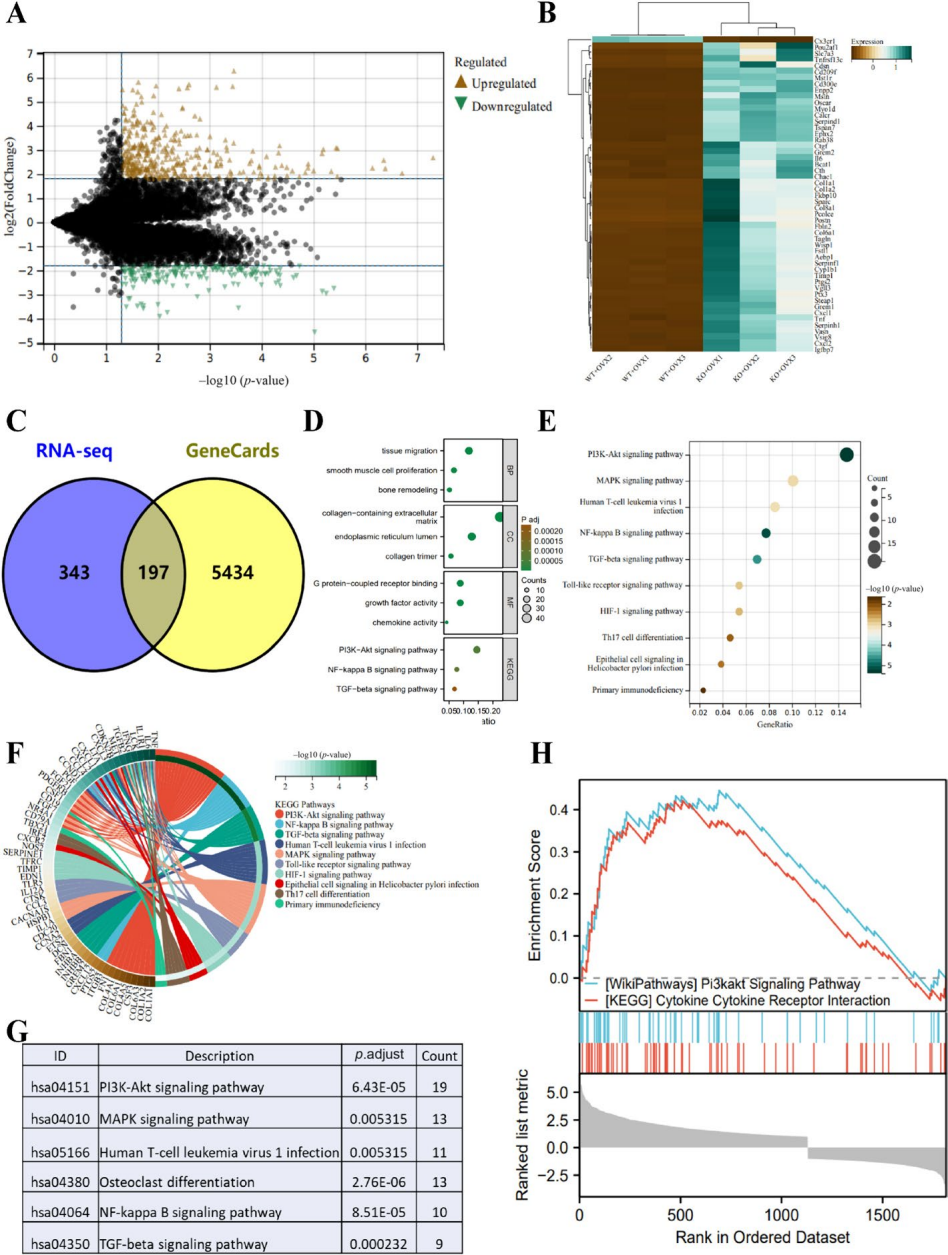
Transcriptional profiling reveals the key pathways influenced by the P2X7 receptor in OP. **A** Volcano plot of differential expression analysis based on transcriptome sequencing; **B** heatmap of selected differentially expressed genes; **C** Venn diagram showing the intersection of genes related to “OP” from the transcriptome sequencing and GeneCards database; **D** scatter plot of GO and KEGG enrichment analysis; **E** scatter plot of KEGG enrichment analysis for relevant pathways; **F** circular plot of KEGG pathway enrichment analysis highlighting the involvement of key genes; **G**, **H** enrichment analysis results of the key gene set using GSEA. Three mice per group were used for transcriptome sequencing

On the basis of the above experiments, P2X7^f/f^; LysM-cre mice and the corresponding OP model were successfully established. Subsequently, we performed transcriptome sequencing and differential analysis on the femoral tissues of the WT + OVX and KO + OVX mice, identifying 540 significantly differentially expressed genes, including 448 upregulated genes and 92 downregulated genes (Fig. 1A; Table S3). The expression patterns of these genes are shown in Fig. 1B, providing a theoretical basis for a better understanding of the impact of P2X7 receptors on the pathogenesis of OP.

Next, we used the GeneCards website to retrieve 5631 genes related to OP and obtained the intersection with our list of 540 differentially expressed genes. This yielded a total of 197 key genes that may be involved in the development of OP under the condition of P2X7 knockout (Fig. 1C).

To gain insights into the functional impact of these genes in the pathogenesis of OP, we conducted a functional enrichment analysis of the biological processes and pathways in which these genes participate using Gene Ontology (GO) and KEGG analysis (Fig. 1D). We found that the PI3K-AKT signaling pathway played an important role (Fig. 1E–G). Furthermore, we performed GSEA functional enrichment analysis on the set of 197 differentially expressed genes, which also revealed significant regulation of the PI3K-AKT signaling pathway (Fig. 1H).

In summary, the collective findings suggest that P2X7 receptors likely influence the pathogenesis of OP through the PI3K-AKT signaling pathway.

### Metabolomic analysis reveals the role of the PI3K-AKT signaling pathway in P2X7-mediated OP

**Fig. 2:**
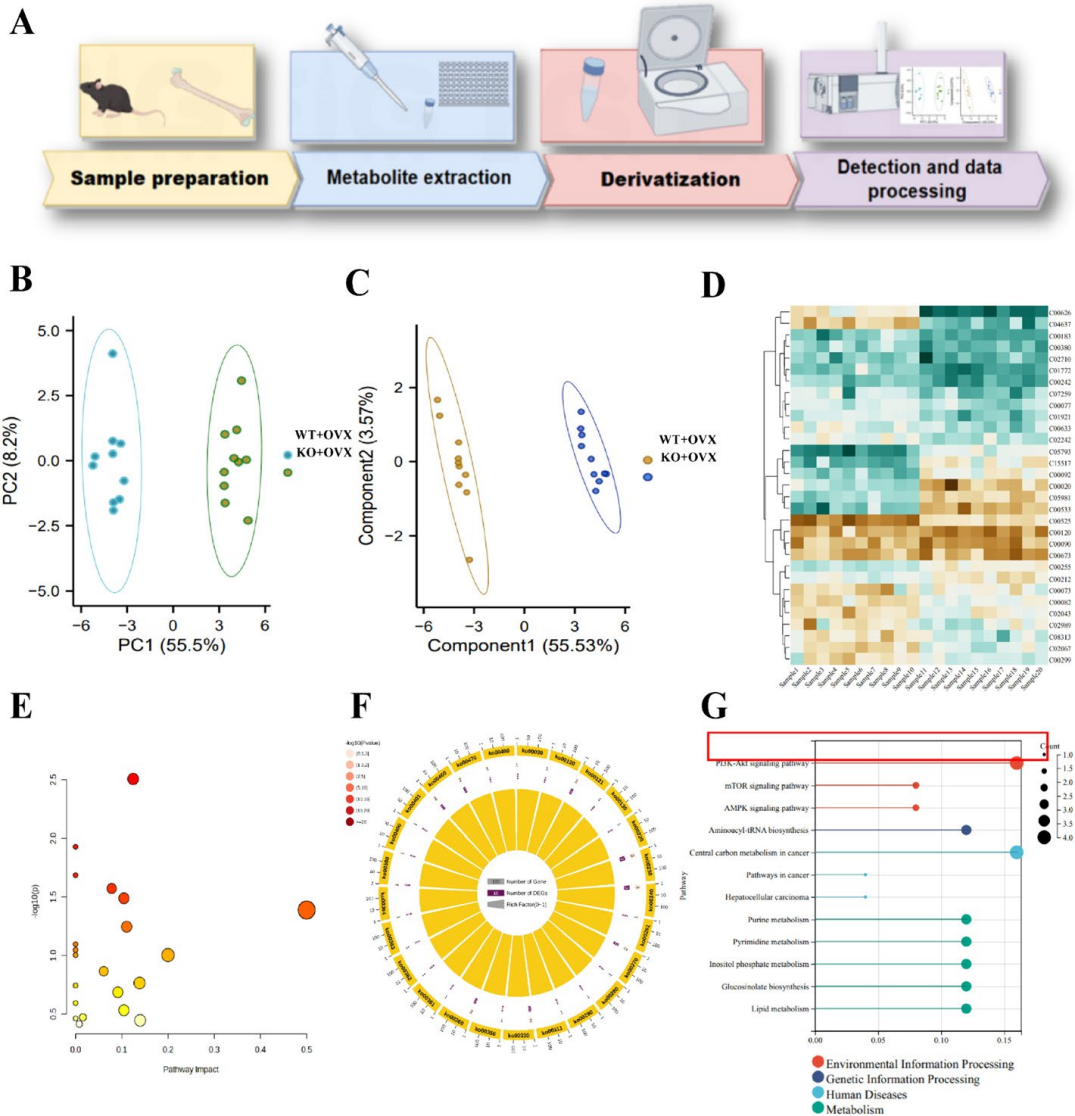
The metabolomic analysis demonstrates altered bone metabolism induced by P2X7 activation. **A** Workflow for LC/MS detection and metabolomic analysis of mouse fecal samples; **B** principal component analysis (PCA) score plot. **C** orthogonal projections to latent structures discriminant analysis (OPLS-DA) score plot. **D** heatmap of 33 differentially regulated metabolites. **E** scatter plot of KEGG enrichment analysis for differentially regulated metabolites; **F** circular plot of KEGG enrichment analysis for differentially regulated metabolites; **G** lollipop plot of KEGG pathway enrichment analysis for differentially regulated metabolites, with larger dots representing more associated metabolites and the red box highlighting the PI3K-AKT signaling pathway with the highest relevance. In total, ten mice were used in each group for the study

Osteoporosis (OP) is a systemic metabolic bone disease. Numerous studies have highlighted the significant role of the gut microbiota and its metabolites in regulating osteoclast formation and bone resorption [50–52]. To comprehensively understand the impact of the P2X7 receptor on the pathogenesis of OP, we conducted further metabolomic analysis on fecal samples from a mouse model, as depicted in Fig. 2A. Metabolites in the samples were analyzed using liquid chromatography-tandem mass spectrometry (LC–MS/MS). Initially, samples underwent methanol treatment for protein precipitation and metabolite extraction, followed by separation using specific liquid chromatography columns.

PCA and orthogonal projections to latent structures-discriminant analysis (OPLS-DA) were employed to generate score plots of the different groups (Fig. 2B, C). Our experimental results clearly showed a distinct separation between the two groups in terms of metabolic profiles. On the basis of these findings, we identified 33 metabolites that displayed significant differences in the P2X7^f/f^; LysM-cre mouse OP model (Table S4). The heatmap of these differentially expressed metabolites is presented in Fig. 2D.

To further explore the potential functions and roles of these differentially expressed metabolites in relation to the crucial role played by P2X7 receptors, enrichment analysis was conducted using the MetaboAnalyst 5.0 database. Detailed information regarding the biological roles of identified metabolites in these pathways was obtained by employing the KEGG and MetaboAnalyst databases. The results revealed significant impacts on several core metabolic pathways, such as the PI3K-Akt-GSK3β signaling pathway. This analysis demonstrated that the PI3K-Akt-GSK3β pathway serves a central role in the regulation of OP by P2X7 receptors (Fig. 2E–G).

Additionally, correlation network analysis was carried out to examine the associations between differentially expressed metabolites and genes identified from transcriptomic sequencing. The visualization heatmap (Fig. S4A) indicated strong correlations between PI (16:0/16:0), Phosphatidylinositol-3,4,5-trisphosphate (PIP3), Nitric oxide (NO), Phosphatidylinositol-4,5-bisphosphate (PIP2), and key differentially expressed genes. These metabolites play crucial roles in the PI3K-AKT-GSK3β signaling pathway, as shown in Fig. S4B. In this pathway, NO activates the phosphorylation of PI3K, which initiates downstream interactions with AKT by phosphorylating PIP2 into PIP3 within the cell membrane.

The results from the metabolomics analysis, along with their correlation with transcriptomics, further support the critical importance of the PI3K-AKT-GSK3β signaling pathway in the development of OP influenced by P2X7.

### The effect of P2X7 receptor on osteoclast differentiation and function

Therefore, we extracted BMM from C57BL/6 mice to observe the role of P2X7 in osteoclast differentiation. The BMM cells were cultured under specific conditions to obtain precursors that could be used for subsequent experiments (Fig. S5A). We validated the extracted BMM cells by flow cytometry, marking them as F4/80^+^ CD11b^+^. The results showed that the percentage of double-positive cells reached 90% (Fig. S5B). We induced osteoclast differentiation using 50 ng/mL M-CSF and RANKL in BMM cells and observed the appearance of differentiated osteoclasts and resorption activity through TRAP staining and pit assays (TRAP absorbance: 0.27 versus 0.55; absorption well area: 0.25 versus 5.91; Fig. S5C-D). Compared with the cells not induced with M-CSF and RANKL, the difference was significant. By performing western blot analysis for MMP-9, CK, and NFATc1 expression in the cells induced or not induced with M-CSF and RANKL, we observed a significant upregulation of these key proteins in the cells treated with M-CSF + RANKL (MMP-9: 1.00 versus 2.49; CK: 1.00 versus 1.98; NFATc1: 1.00 versus 2.31; Fig. S5E). These experimental results confirmed that the extracted BMM cells possessed the necessary conditions for studying osteoclast differentiation.

**Fig. 3:**
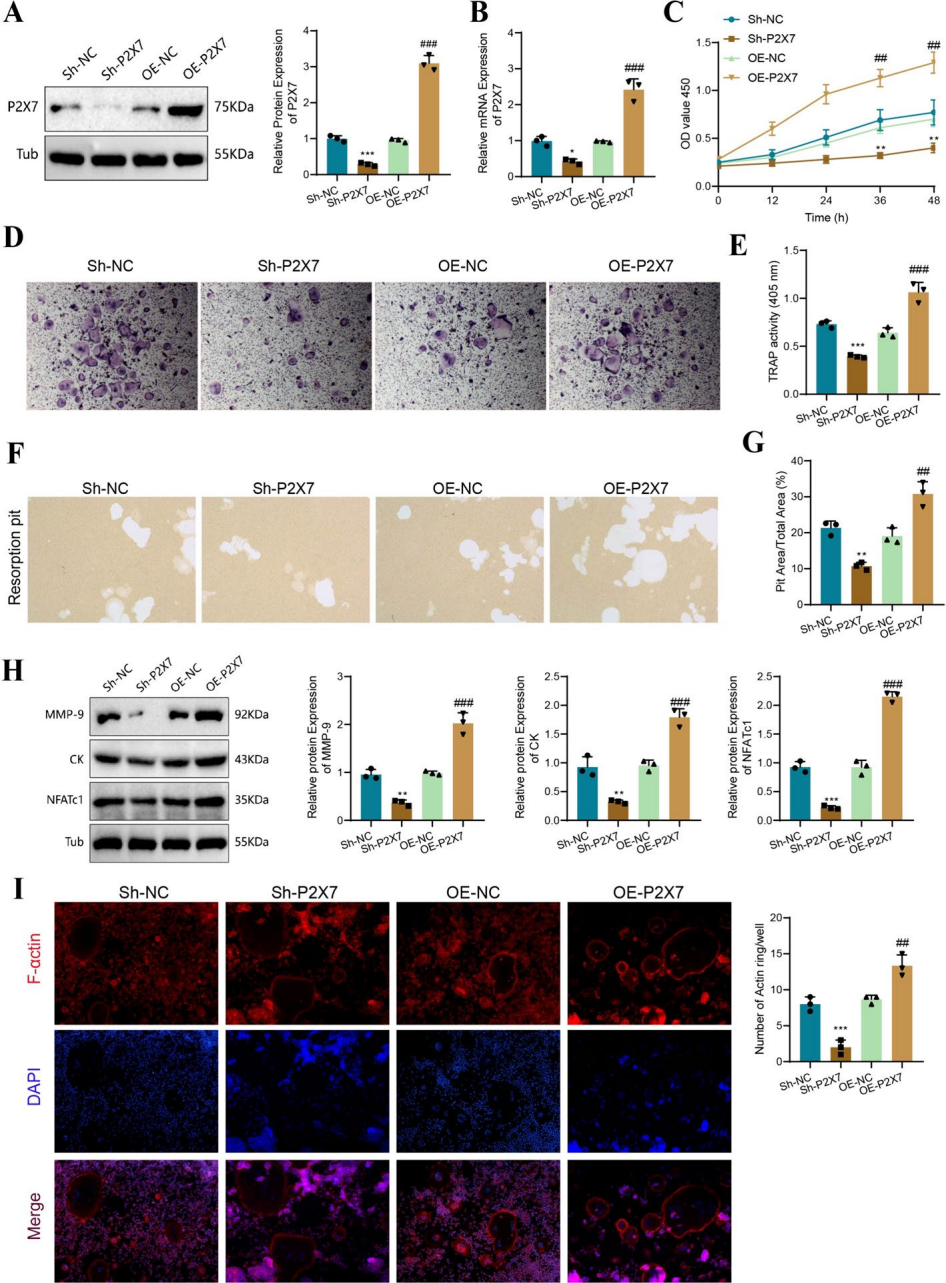
Impact of P2X7 on osteoclast differentiation and resorption. **A** Western Blot analysis of P2X7 protein expression levels and grayscale value statistics in different cell groups; **B** RT-qPCR analysis of P2X7 mRNA expression levels and statistics in different cell groups; **C** CCK-8 assay to measure cell proliferation at 0, 12, 24, 36, and 48 h in different groups; **D** TRAP staining of cells in different groups; **E** statistics of TRAP activity; **F** measurement of resorption pits in different cell groups; **G** statistics of the percentage of area covered by resorption pits in different cell groups; **H** western blot analysis of protein expression levels and grayscale values of MMP-9, CK, and NFATc1 in different cell groups; **I** F-Actin ring staining results and statistics of immunofluorescence-positive cell counts. Bar = 50 μm. **P < 0.01, ***P < 0.001 compared with Sh-NC; ^##^*P* < 0.01, ^###^*P* < 0.001, compared with OE-NC. Cell experiments were repeated three times

Subsequently, we silenced or overexpressed P2X7 in the extracted BMM cells through lentivirus transfection. Western blot and PCR analyses revealed that Sh-P2X7 significantly reduced the protein and mRNA levels of P2X7 (relative protein level: 1.00 versus 0.29; relative mRNA level: 0.99 versus 0.42), while OE-P2X7 upregulated P2X7 expression (relative protein level: 0.95 versus 3.10; relative mRNA level: 0.97 versus 2.42; Fig. 3A, B).

Furthermore, we conducted CCK-8 experiments to investigate the effects of P2X7 on cell viability and proliferation. The results showed that the OE-P2X7 group exhibited higher activity and proliferation compared with the OE-NC group (0.70 versus 1.29), whereas the Sh-P2X7 group showed reduced activity and proliferation (0.77 versus 0.40), indicating the core role of P2X7 in osteoclast precursor cell proliferation (Fig. 3C). After the Sh-P2X7 treatment, the number of osteoclasts significantly decreased compared with the Sh-NC group, while the OE-P2X7 group demonstrated the opposite effect, with a significant increase in the number of osteoclasts.

We conducted TRAP staining and immunofluorescence staining experiments to investigate the effects of P2X7 on osteoclast differentiation and function. The results of TRAP staining showed that both Sh-NC and OE-NC groups exhibited cells with a red–purple color. However, in the Sh-P2X7 group, the staining of osteoclasts was significantly reduced, appearing lighter in color, while the OE-P2X7 group showed an increase in stained cells with a deeper purple color, indicating a decrease in osteoclast differentiation in the Sh-P2X7 group and an increase in osteoclast differentiation in the OE-P2X7 group (Fig. 3D–F). The resorption pit assay results showed a similar proportion of resorption pits in the Sh-NC and OE-NC groups. However, in the Sh-P2X7 group, the number and area of resorption pits were significantly reduced, whereas, in the OE-P2X7 group, they were significantly increased, suggesting the impact of P2X7 on osteoclast resorption activity (Fig. 3G).

Next, we used western blot analysis to evaluate the expression of key proteins involved in osteoclast differentiation, including MMP9, CK, and NFATc1. Compared with the Sh-NC group, Sh-P2X7 significantly downregulated the expression levels of these factors, whereas OE-P2X7 upregulated their expression levels (Fig. 3H). F-actin rings, composed of microfilaments (F-actin) in the shape of a ring structure, appear during the differentiation of macrophages into osteoclasts and are indicative of bone resorption activity. Thus, we assessed the expression of F-actin and observed that the Sh-P2X7 group exhibited weaker fluorescence staining and no formation of circular F-actin, while the OE-P2X7 group showed significantly higher fluorescence intensity and clear circular staining, indicating enhanced resorption activity of osteoclasts in the OE-P2X7 group (Fig. 3I).

Collectively, these results demonstrate that the P2X7 receptor directly influences osteoclast differentiation and resorption activity.

### The regulation of PI3K-AKT-GSK3β signaling by P2X7 receptor

**Fig. 4:**
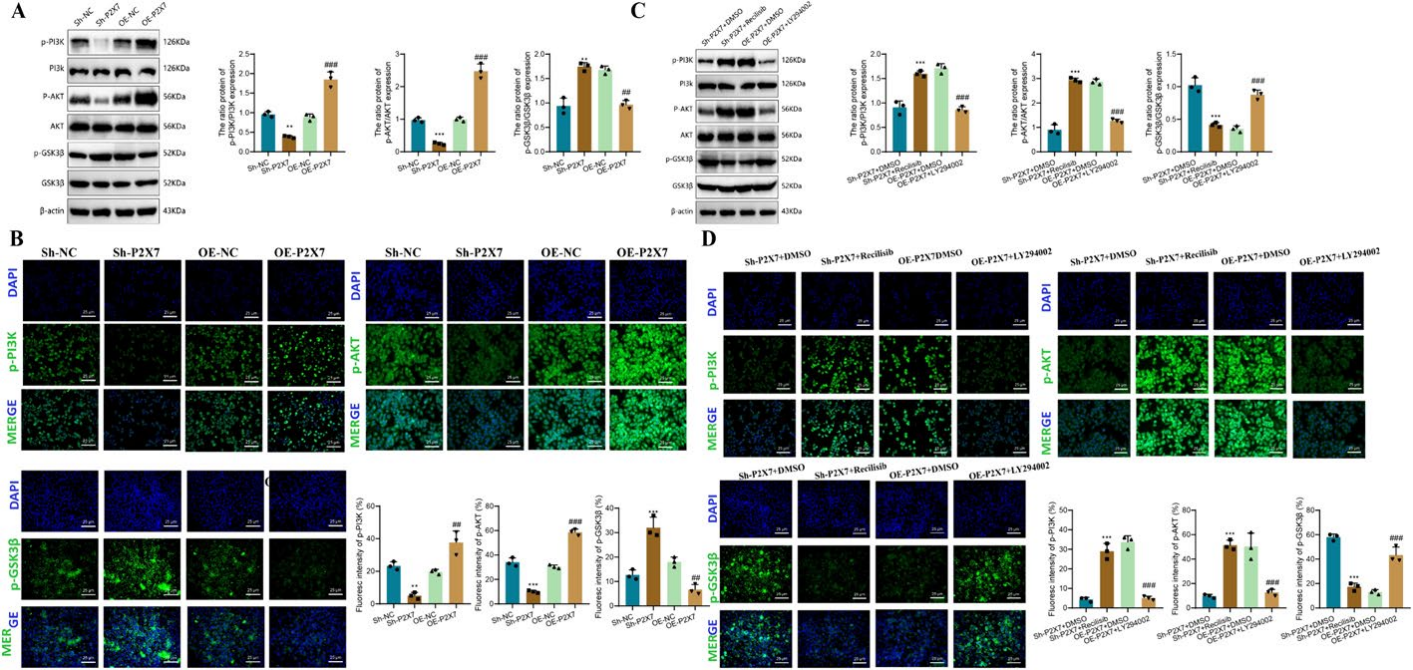
Regulatory role of P2X7 on the PI3K-Akt-GSK3β pathway. **A** Western blot analysis and grayscale value statistics of key factors in the PI3K-Akt-GSK3β signaling pathway phosphorylation levels in BMM cells transfected with P2X7 siRNA or overexpressed P2X7 lentivirus; **B** immunofluorescence staining and statistics of positive area percentage of key factors in the PI3K-Akt-GSK3β signaling pathway in different cell groups; **C** western blot analysis and grayscale value statistics of key factors in the PI3K-Akt-GSK3β signaling pathway phosphorylation levels in BMM cells transfected with P2X7 siRNA or overexpressed P2X7 lentivirus, and treated with activators or inhibitors of the PI3K-AKT-GSK3β signaling pathway; **D** immunofluorescence staining and statistics of positive area percentage of key factors in the PI3K-Akt-GSK3β signaling pathway in different cell groups. Bar = 50 μm. For (**A**, **B**), **P < 0.01, ***P < 0.001 compared with the Sh-NC group; ##*P* < 0.01, ###*P* < 0.001 compared with the OE-NC group. For (**C**, **D**), **P < 0.01, ***P < 0.001 compared with Sh-P2X7 + DMSO group; ^##^*P* < 0.01, ^###^*P* < 0.001 compared with OE-P2X7 + DMSO group

First, we performed a western blot analysis to determine the expression levels of key factors in the PI3K-AKT-GSK3β signaling pathway. We observed a significant decrease in the phosphorylation levels of PI3K and AKT in Sh-P2X7 cells (p-PI3K: 0.96 versus 0.38; p-AKT: 0.97 versus 0.25) while the phosphorylation level of GSK3β increased (0.94 versus 1.74). Conversely, in OE-P2X7 cells, we observed a significant upregulation of PI3K and AKT phosphorylation levels (p-PI3K: 0.88 versus 1.85; p-AKT: 1.00 versus 2.47), and GSK3β showed lower levels of phosphorylation (1.68 versus 0.96) (Fig. 4A). The results obtained from immunofluorescence staining of the phosphorylated key factors were consistent with the western blot analysis (Fig. 4B).

Having observed the impact of P2X7 on the phosphorylation levels of key factors in the PI3K-AKT-GSK3β signaling pathway, we further investigated the relationship between P2X7 and PI3K-AKT-GSK3β by treating cells with Recilisib, a pathway activator, and LY294002, an inhibitor, at a concentration of 10 μM. Specifically, we treated cells with these compounds after silencing or overexpressing P2X7 to gain deeper insight into their relationship. We found that Recilisib attenuated the inhibitory effect of Sh-P2X7 on the activation of the PI3K-AKT-GSK3β signaling pathway. Compared with the Sh-P2X7 + DMSO group, the expression levels of p-PI3K and p-AKT increased, while p-GSK3β expression decreased. Similarly, in OE-P2X7 cells, LY294002 inhibited the activation of the PI3K-AKT-GSK3β signaling pathway. Specifically, compared with the OE-P2X7 + DMSO group, the expression levels of p-PI3K and p-AKT decreased, while p-GSK3β expression increased (Fig. 4C). The results obtained from cell immunofluorescence staining further supported these findings (Fig. 4D).

Our results provide direct evidence of the impact of P2X7 expression on the activation of the PI3K-AKT-GSK3β signaling pathway in BMM cells.

### P2X7 receptor regulates osteoclast differentiation and resorption through the PI3K-AKT-GSK3β pathway

**Fig. 5:**
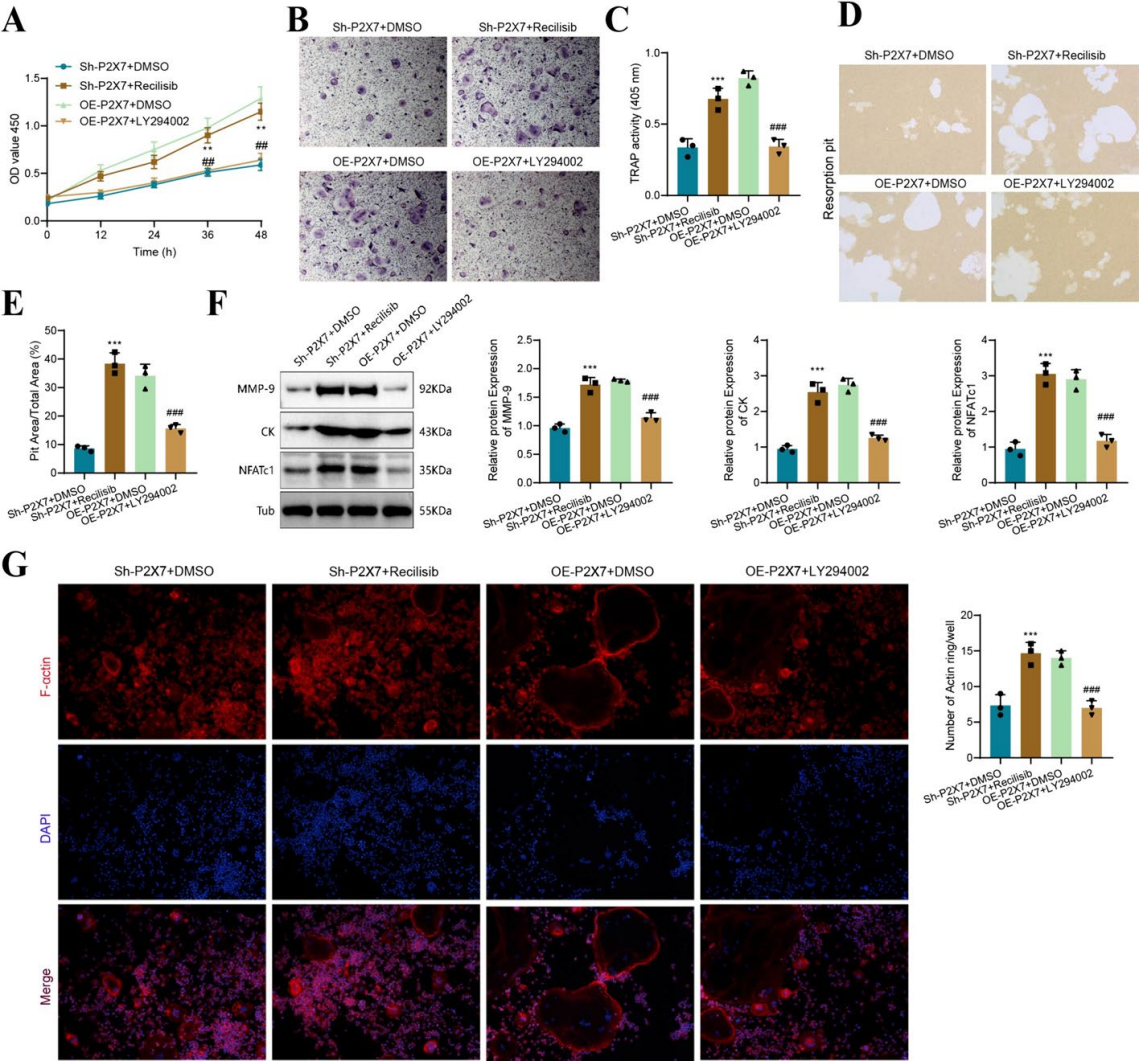
The influence of P2X7 receptor regulation on PI3K-Akt-GSMP3β and osteoclast function. **A** CCK-8 assay measured the absorbance of cells in various groups at 0, 12, 24, 36, and 48 h; **B** representative images of TRAP staining results; **C** spectrophotometer measured the absorbance of TRAP-stained cells at a wavelength of 405 nm; **D** resorption pit assay evaluated the differentiation and absorption ability of precursor cells in each group; **E** bar graph presenting the percentage of resorption pit area for each group; **F** western blot analysis determined the expression levels and grayscale values of MMP-9, CK, and NFATc1 in cells from each group; **G** bar graph illustrating the results of F-actin ring staining and the percentage of positive area. Bar = 50 μm. Compared with the Sh-P2X7 + DMSO group, **P < 0.01, ***P < 0.001; compared with the OE-P2X7 + DMSO group, ^##^*P* < 0.01, ^###^*P* < 0.001

On the basis of the essential role of the PI3K-AKT-GSK3β signaling pathway in osteoclast differentiation, formation, and absorption, as well as the known impact of P2X7 on this pathway, we propose the hypothesis that P2X7 affects the differentiation of osteoclast precursor cells into osteoclasts by regulating the PI3K-AKT-GSK3β signaling pathway. To test this hypothesis, we treated BMM cells with Sh-P2X7 and OE-P2X7 interventions, along with activators and inhibitors of the PI3K-AKT-GSK3β pathway, to observe the differentiation and absorption of cells in each group. As a control, the Sh-P2X7 and OE-P2X7 groups were treated with DMSO. Initially, we assessed the proliferative activity of the cells in each group using CCK-8. We found that the proliferation activity of the Sh-P2X7 + recilisib group increased compared to the Sh-P2X7 + DMSO group (0.59 versus 1.15), while the proliferation activity of the OE-P2X7 + LY294002 group decreased compared to the OE-P2X7 + DMSO group (1.29 versus 0.64; Fig. 5A). Through microscopic observation and cell counting, it was discovered that the number of TRAP-stained cells in the Sh-P2X7-recilisib group significantly increased compared with the Sh-P2X7 group (Fig. 5B, C). This finding indicates that recilisib markedly enhances the absorbance in Sh-P2X7 (0.34 versus 0.68). On the other hand, the absorbance in the OE-P2X7 + LY294002 group significantly decreased compared with the OE-P2X7 group (0.82 versus 0.34; Fig. 5B, C), suggesting that LY294002 can inhibit the promotion of osteoclast differentiation by OE-P2X7.

Furthermore, we assessed the active absorption capacity of osteoclasts in each group using pit resorption assays. We found that the area occupied by resorption pits significantly increased in the Sh-P2X7 + recilisib group compared with the Sh-P2X7 + DMSO group (3.50 versus 13.94), indicating enhanced absorption capability of active osteoclasts. Conversely, the promotion of osteoclast absorption by OE-P2X7 was inhibited by the PI3K-AKT-GSK3β pathway inhibitor LY294002 (14.96 versus 5.01; Fig. 5D, E). MMP-9, CK, and NFATc1, proteins associated with osteoclast differentiation, were downregulated in BMM cells of the Sh-P2X7 + DMSO group (MMP-9: 0.96 versus 1.72; CK: 0.95 versus 2.54; NFATc1: 0.95 versus 3.05) but significantly increased by recilisib. In contrast, their expression was upregulated in BMM cells of the OE-P2X7 + DMSO group but significantly inhibited by LY294002 (MMP-9: 1.79 versus 1.14; CK: 2.75 versus 1.26; NFATc1: 2.90 versus 1.17; Fig. 5F). F-actin fluorescence staining results supported the above findings, with recilisib significantly increasing the expression of low-expressed F-actin with low fluorescence intensity in the Sh-P2X7 group, while LY294002 inhibited the expression of significantly fluorescent, circular F-actin in the OE-P2X7 + DMSO group (Fig. 5G).

Taken together, these results elucidate that the P2X7 receptor affects the differentiation and absorption of osteoclasts through the PI3K-AKT-GSK3β signaling pathway.

### P2X7 receptor activates PI3K-AKT-GSK3β signaling to regulate osteoclast differentiation and resorption in OP

In in vitro experiments with BMM cells, the pivotal role of the P2X7 receptor in osteoclast differentiation, as well as the critical involvement of the PI3K-AKT-GSK3β signaling pathway, was evident. To deepen our understanding of the role of the PI3K-Akt-GSK3β signaling pathway in the pathogenesis of osteoporosis, we used WT mice and P2X7^f/f^; LysM-cre mice as experimental subjects. Initially, we performed OVX surgery to simulate the onset of osteoporosis and categorized the mice into four groups: WT + Sham, WT + OVX, KO + OVX, and KO + OVX + recilisib. In the KO + OVX + recilisib group, mice received daily intraperitoneal injections of recilisib starting the day after the surgery.

**Fig. 6:**
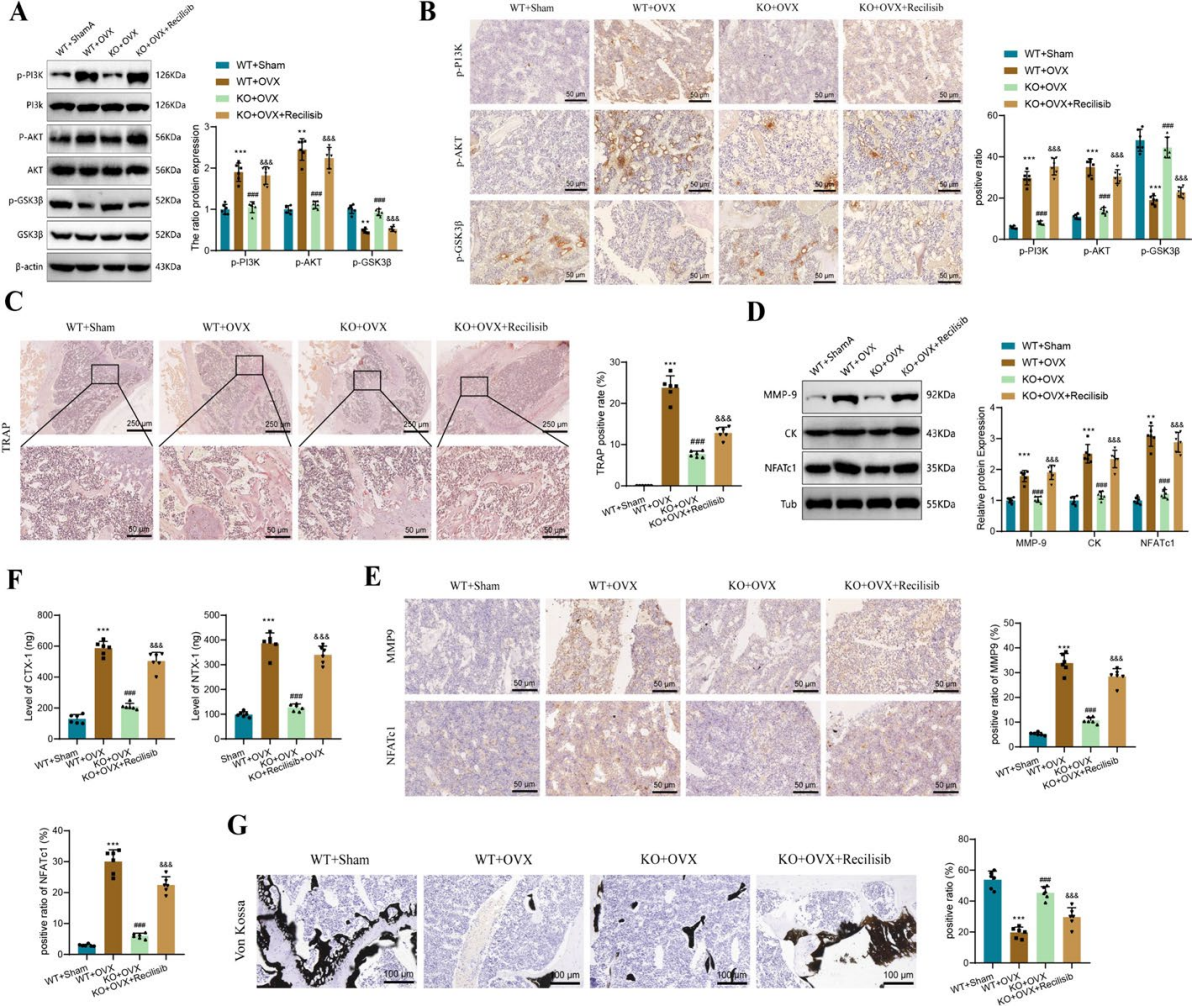
The regulatory effect of P2X7 receptor via the PI3K/AKT/GSK3β signaling pathway in vivo. **A** Western blot analysis measured the phosphorylation levels and grayscale values of P13K, AKT, and GSK3β proteins in mouse femur tissue from each group; **B** immunohistochemistry staining assessed the expression and positive area percentage of p-P13K, p-AKT, and p-GSK3β in mouse femur tissue of each group; **C** bar graph presenting the number of TRAP-positive cells in mouse femur tissue sections stained with TRAP; **D** western blot analysis determined the expression levels and band grayscale values of MMP9, NFATc1, and CK proteins in mouse femur tissue from each group; **E** immunohistochemistry staining assessed the expression and positive area percentage of MMP9 and NFATc1 in mouse femur tissue; **F** ELISA measured the expression levels of CTX and NTX in mouse serum; **G** Von Kossa staining assessed the calcium salt deposition in mouse femur tissue sections. Bar = 100 μm. Compared with the WT + Sham group, ***P < 0.001; compared with the WT + OVX group, ^###^*P* < 0.001; compared with the KO + OVX group, ^&&&^*P* < 0.001

To investigate the impact of the P2X7 receptor on the PI3K-AKT-GSK3β signaling pathway in vivo, we first examined the phosphorylation of key factors in the pathway in femoral bone tissue using western blot analysis. The results showed that compared with the WT + Sham group, the levels of phosphorylated PI3K and AKT in femoral bone tissue proteins were upregulated in the WT + OVX group (p-PI3K: 1.00 versus 1.90; p-AKT: 1.05 versus 1.82), while the phosphorylation level of GSK3β was downregulated (1.00 versus 0.47). In contrast, in the KO + OVX group, the activation of the pathway was suppressed compared to the WT + OVX group, characterized by inhibited phosphorylation of PI3K and AKT (p-PI3K: 1.90 versus 1.05; p-AKT: 2.45 versus 1.11) and increased phosphorylation of GSK3β (0.47 versus 0.93). Following daily intraperitoneal injection of recilisib, the PI3K-AKT-GSK3β signaling pathway was reactivated (Fig. 6A). Immunohistochemistry staining provided results similar to those of western blot analysis (Fig. 6B).

In our in vitro experiments, we observed that the P2X7 receptor could induce the differentiation of BMM cells into osteoclasts and enhance their absorptive function. Our validation demonstrated that this effect was achieved through the modulation of the PI3K-AKT-GSK3β pathway. To further confirm our findings, we conducted relevant tests on osteoclast differentiation and absorption in an animal model.

The histological analysis of femur tissue sections from different groups of mice stained with TRAP revealed significant findings. In the WT + Sham group, the femur tissue exhibited well-preserved morphology with minimal TRAP staining, as evidenced by the absence of TRAP-stained cells upon statistical analysis. Conversely, in the WT + OVX group, sections showed noticeable red-stained cells. Comparatively, in the KO + OVX group, TRAP staining was significantly suppressed, leading to a marked reduction in the number of stained cells (23.81 versus 7.69), suggesting a protective effect against osteoporosis in P2X7 gene knockout mice. However, daily administration of recilisib attenuated the protective effect of P2X7 knockout, as observed by an increase in the number of stained cells in the KO + OVX + recilisib group (7.69 versus 12.84), reaching levels similar to WT + OVX, indicating a significant difference compared with the KO + OVX group (Fig. 6C).

Subsequently, we examined the expression levels of key proteins involved in osteoclast differentiation, including MMP9, CK, and NFATc1, in the femur tissues of model mice in each group. The western blot results revealed that in the WT + OVX group, the expression of the aforementioned proteins was significantly upregulated compared to the WT + Sham group (MMP-9: 1.00 versus 1.79; CK: 1.00 versus 2.51; NFATc1: 1.00 versus 3.08), indicating an increased degree of osteoclast differentiation. In contrast, in the femur tissues of KO + OVX mice, the expression of these proteins was suppressed compared with the WT + OVX group, suggesting an inhibitory effect of P2X7 knockout on osteoclast differentiation. However, treatment with recilisib reversed this inhibitory effect, as evidenced by the upregulation of MMP9, CK, and NFATc1 in the KO + OVX + recilisib group compared with the KO + OVX group (MMP-9: 1.03 versus 1.91; CK: 1.17 versus 2.34; NFATc1: 1.21 versus 2.88), reaching levels similar to the WT + OVX group (Fig. 6D). Immunohistochemical staining results for MMP9 and NFATc1 corresponded to the western blot findings (Fig. 6E).

These results collectively demonstrate the impact of the P2X7 receptor on osteoclast differentiation. To further assess osteoclast absorption and bone metabolism, we used ELISA to measure the levels of C-terminal and N-terminal crosslinked peptides (CTX and NTX, respectively) in the mice’s serum. CTX and NTX are degradation products of collagen that are released into the bloodstream during bone resorption. Increased activity of osteoclasts and occurrence of absorption can be detected by higher levels of CTX and NTX in the serum, making them critical markers for OP and fracture risk assessment [53]. In our study, we observed a significant increase in serum levels of CTX and NTX in WT + OVX mice compared with the WT + Sham group (CTX: 129.33 versus 587.17; NTX: 98.83 versus 386.00). This suggests that active osteoclasts are undergoing resorption, releasing metabolites into the serum. In contrast, the serum levels of CTX and NTX in KO + OVX mice decreased compared with the WT + OVX group, indicating that P2X7 deletion reduced osteoclast activity and resorption, providing a protective effect against osteoporosis. Furthermore, compared with the KO + OVX group, the KO + OVX + recilisib group showed a significant increase in serum CTX and NTX levels (CTX: 209.83 versus 503.50; NTX: 127.00 versus 339.83), suggesting that recilisib injection treatment reversed the protective effect of P2X7 deletion (Fig. 6F).

The activity of osteoclasts is dynamically balanced with bone mineralization, and increased osteoclast activity leads to a decrease in calcium salt content in the bone. On the basis of this, we used Von Kossa staining to assess the level of calcium salt deposition in the femoral bone tissue of the different groups and evaluate osteoclast absorption. The results showed that compared with the WT + Sham group, the degree of calcium salt deposition was significantly reduced in the WT + OVX group (53.83 versus 19.67). This reduction was alleviated in the KO + OVX group, and recilisib treatment further decreased calcium salt deposition in the femoral bone tissue (45.33 versus 29.67; Fig. 6G).

These results indicate that the P2X7 receptor positively regulates the phosphorylation of the PI3K-AKT-GSK3β signaling pathway in vivo. Through this regulation, osteoclast differentiation and absorption are activated.

### P2X7 receptor regulates osteoclasts and contributes to OP progression

In both in vitro and in vivo experiments, we have demonstrated the impact of P2X7 receptors on the differentiation and resorption of osteoclasts, suggesting a potential mechanism by which P2X7 influences OP. To validate our hypothesis, we performed morphological examinations on a mouse model of OP established in vivo to observe the direct impact of P2X7 on the progression of OP in mice.

**Fig. 7:**
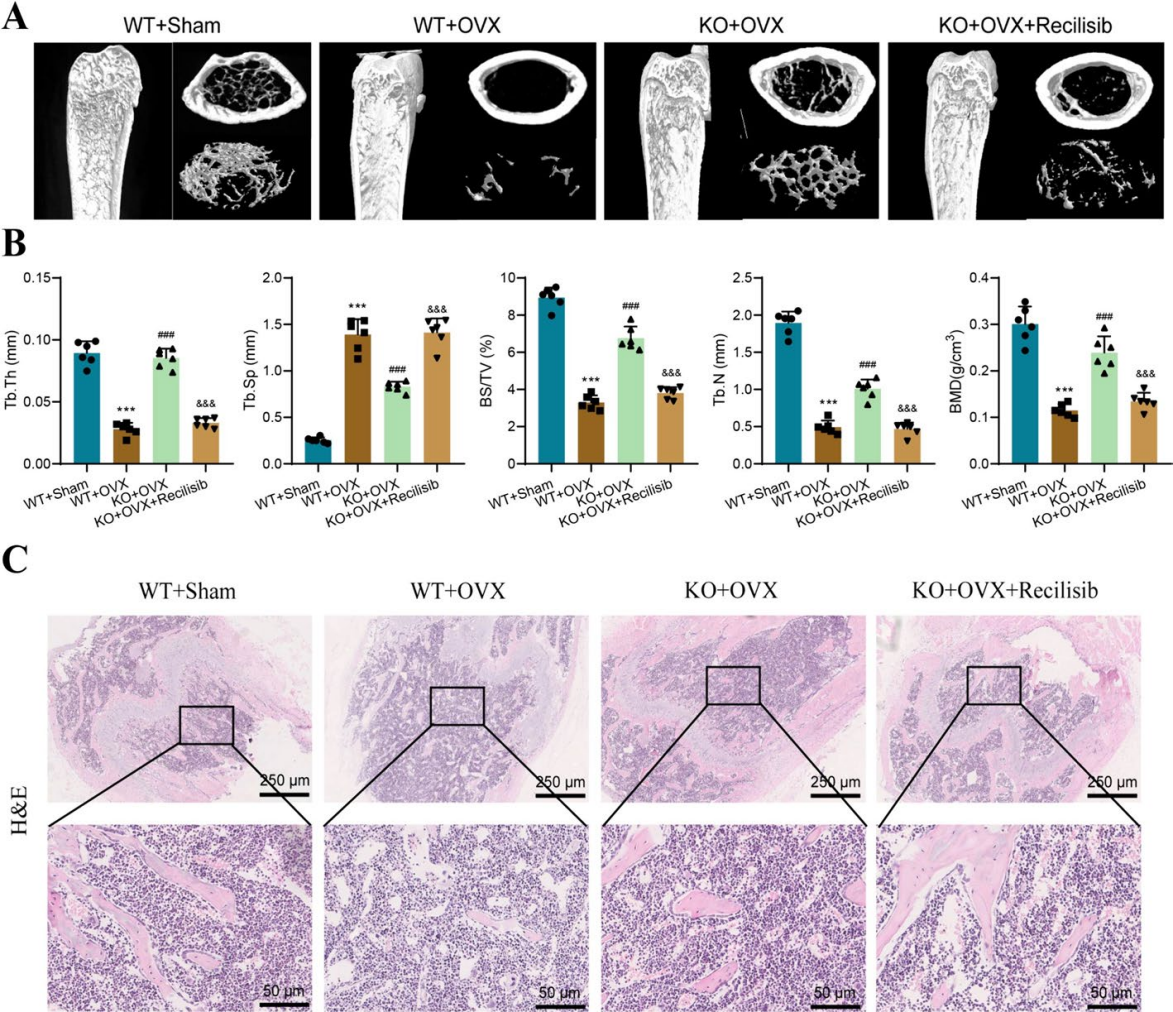
The impact of the P2X7 receptor on the progression of OP in mice is mediated through the regulation of the PI3K/AKT/GSK3β pathway. **A** Representative images of mouse femur micro CT in different groups; **B** evaluation of micro CT parameters (Tb.Th, Tb.Sp, BV/TV, Tb.N, BMD); **C** HE staining results of mouse femoral tissue sections in different groups. Bar = 400 μm and 100 μm. Compared with the WT + Sham group, ***P < 0.001; compared with the WT + OVX group, ^###^*P* < 0.001; compared with the KO + OVX group, ^&&&^*P* < 0.001. Each group consists of six mice

Firstly, we utilized a micro-CT instrument to quantitatively analyze changes in bone microstructure at a high resolution. The micro-CT scan results presented in Fig. 7A show that, compared with the WT + Sham group, mice in the WT + OVX group exhibited significant decreases in bone density, increased trabecular spacing, and reduced BMD. Conversely, mice in the KO + OVX group displayed a certain degree of protective effect, implying the potential role of P2X7 receptors in OP. However, after treatment with recilisib, the protective effect of P2X7 knockout disappeared (Fig. 7B).

Furthermore, HE staining was used to observe and analyze tissue morphology. The results of HE staining revealed that the bone tissue in the WT + Sham group exhibited regular and organized trabecular arrangement with smaller marrow cavities. In contrast, the WT + OVX group displayed more micro-damage and morphological changes in the bone tissue, such as enlarged marrow cavities, increased adipose tissue infiltration, thinning of the cortical bone, and sparse and disordered trabecular arrangement. The KO + OVX group showed a reduction in tissue damage and morphological changes compared with the WT + OVX group. However, the protective effect of P2X7 knockout disappeared after intraperitoneal injection of recilisib, and the bone tissue damage was similar to that of the WT + OVX group (Fig. 7C).

In conclusion, our results reveal that P2X7 receptors positively regulate the formation and function of osteoclasts, thereby influencing the progression of OP by affecting the PI3K-Akt-GSK3β signaling pathway. This provides further molecular understanding of OP and potential therapeutic targets.

## Discussion

In recent decades, the incidence of OP has been increasing, posing significant challenges to global health and socioeconomic systems [8, 54, 55]. OP is a chronic skeletal disorder characterized by reduced bone tissue quality and density, which leads to an increased risk of bone fragility and fractures [56–58]. The development of OP is influenced by various factors, including genetic, environmental, and lifestyle factors [59–61]. OP predisposes patients to increased fracture risk, particularly in elderly individuals, further elevating mortality rates. Therefore, conducting thorough research on OP is crucial for better understanding and management [62]. In the pathogenesis of OP, the imbalance between osteoclast formation and bone resorption is considered a major mechanism [63, 64].

PI3K is a lipid kinase that induces AKT phosphorylation, playing a crucial role in responding to extracellular signals, regulating cell survival, and angiogenesis. GSK3β, considered a key downstream factor in the PI3K-AKT signaling pathway, is phosphorylated by PI3K-AKT, leading to inactivation at the Ser9 site. The activation of PI3K-AKT-GSK3β is closely associated with caspase-3 activation and apoptotic signals, believed to be involved in apoptosis of bone cells in bone disease research [28].

The significance of the P2X7 receptor and PI3K-Akt-GSK3β signaling pathway in OP has drawn considerable attention [22, 65, 66]. The P2X7 receptor is an ion channel receptor expressed in bone marrow stromal cells, osteoclasts, and osteoblasts, which can modulate osteoclast formation and bone resorption mechanisms by regulating intracellular calcium concentration and activating PI3K-Akt-GSK3β signaling pathway [65, 67–69]. However, the exact mechanisms of action of the P2X7 receptor and PI3K-Akt-GSK3β signaling pathway in the development of OP still remain controversial and unclear [31, 32].

Through the combined analysis of transcriptomics and metabolomics, we discovered the significant impact of P2X7 receptor activation on osteoclast formation and bone resorption through the PI3K-Akt-GSK3β signaling pathway. Transcriptomic data indicated that P2X7 receptor activation enhances the expression of osteoclast-related genes, thereby increasing the ability of osteoclasts to form and resorb bone. Metabolomic data revealed the influence of P2X7 receptor activation on the metabolic profile of osteoclasts through the PI3K-Akt-GSK3β signaling pathway, further confirming its important role in regulating bone resorption.

**Fig. 8:**
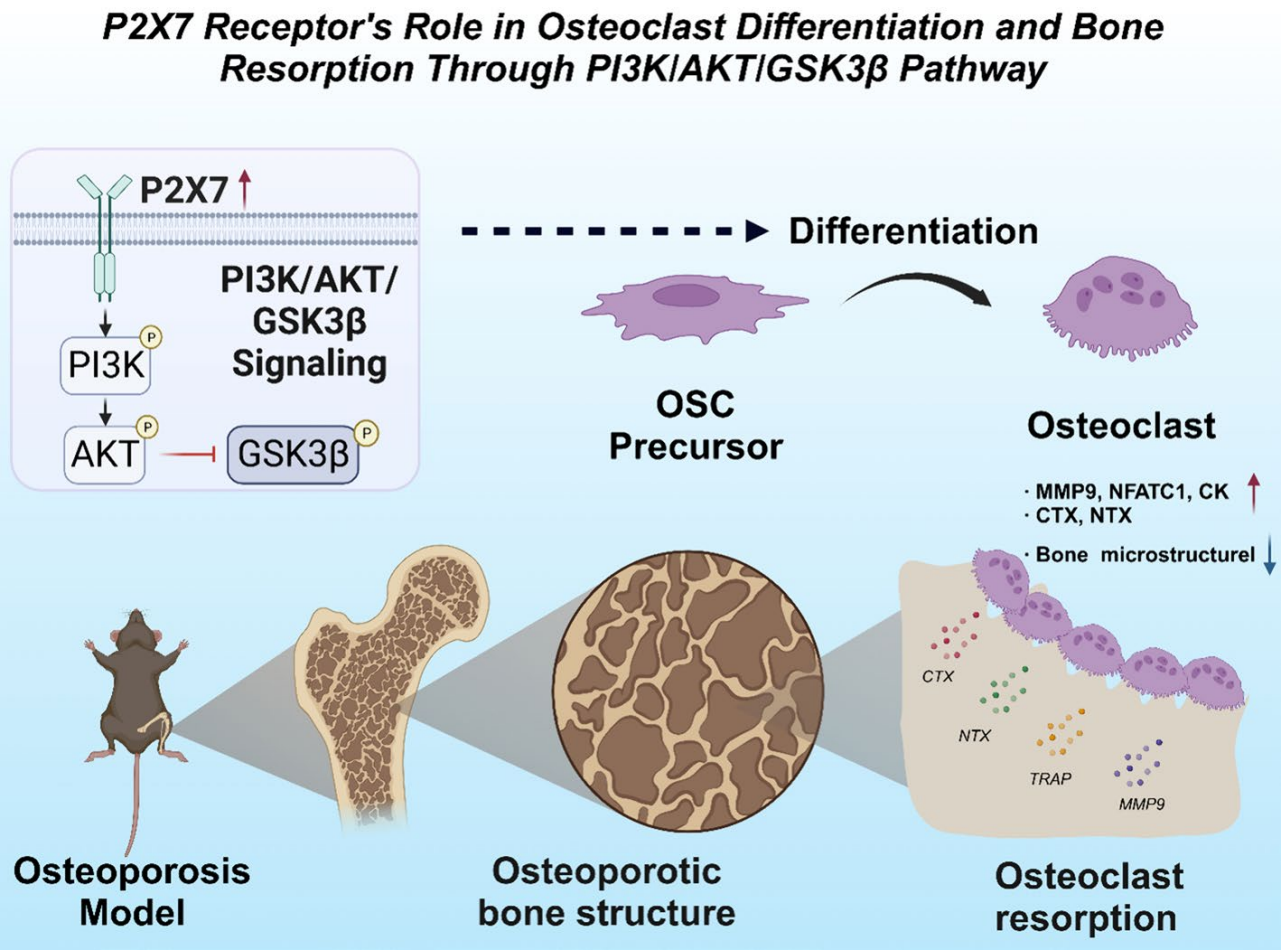
The P2X7 receptor mediates osteoclast differentiation and accelerates bone absorption in OP through the regulation of the PI3K/AKT/GSK3β pathway

To validate the influence of P2X7 receptor activation on osteoclast differentiation and bone resorption capacity through the PI3K-Akt-GSK3β signaling pathway, we conducted in vitro cell experiments. The results showed that when the PI3K-Akt-GSK3β signaling pathway was activated, the P2X7 receptor positively regulated osteoclast formation, playing a critical role in the occurrence and development of OP (Fig. 8). This finding is consistent with some previous studies, but we provide more specific and in-depth evidence.

Although this study has made significant findings and breakthroughs, there are still limitations and shortcomings. Firstly, there may be biases in the experimental design, such as sample selection bias and inconsistency in experimental conditions. Secondly, the sample size of this study is relatively small, which may affect the reliability and generalizability of the results. Additionally, the methods and techniques used in data analysis need to be improved to enhance the accuracy and interpretability of the results. These limitations and shortcomings may have an impact on our interpretation of the research results and their application in clinical settings. Therefore, in future research, we need to carefully address these issues and conduct larger-scale, multicenter studies to further validate and expand the conclusions of this study.

The results of this study have scientific and clinical value in elucidating the pathogenesis and treatment of OP. Firstly, by elucidating the key roles of the P2X7 receptor and the PI3K-Akt-GSK3β signaling pathway in regulating bone resorption, we have further understood the molecular mechanisms underlying the development of OP. Secondly, our research results suggest that activation of the P2X7 receptor and PI3K-Akt-GSK3β signaling pathway may serve as a new therapeutic strategy, providing new targets and approaches for the prevention and treatment of OP.

The results of this study have important applications and future research directions. By further studying the regulatory mechanisms of the P2X7 receptor and PI3K-Akt-GSK3β signaling pathway, as well as developing activators and inhibitors of these signaling pathways, we can optimize and improve the relevant treatment strategies and provide new options for the prevention and treatment of OP.

In summary, this study, through the combined analysis of transcriptomics and metabolomics, reveals the essential roles of the P2X7 receptor and PI3K-Akt-GSK3β signaling pathway in the development of OP. Despite the existence of some limitations and shortcomings, the results of this study have significant scientific and clinical value in our understanding of the pathogenesis of OP and the development of new treatment strategies. Future research should continue to explore the regulatory mechanisms of these signaling pathways and further validate and improve relevant treatment strategies to overcome limitations and enhance applicability.

### Supplementary Information

Additional file 1. Figure S1. Flowchart of in vivo experimental procedure in the OP mouse model.

Additional file 2. Figure S2. Efficiency detection of silencing lentivirus sequence and verification of lentivirus overexpression effect. **A** Western blot detection of P2X7 expression levels and histogram of grayscale values in each group of cells; **B** RT-qPCR detection of P2X7 mRNA levels in each group of cells; **C** Western blot detection of P2X7 expression levels and histogram of grayscale values in each group of cells; **D** RT-qPCR detection of P2X7 mRNA levels in each group of cells. Compared to the Sh-NC or OE-NC group, ***P < 0.001. Cell experiments were repeated three times.

Additional file 3. Figure S3. Successful validation of OP model in c57BL/6 mice and KO-P2X7 mice. **A** Western blot detection and histogram of grayscale values of P2X7 expression in femoral tissues of WT and KO mice. **B** RT-qPCR detection and histogram of mRNA levels of P2X7 expression in femoral tissues of mice. **C** Western blot detection and histogram of grayscale values of P2X7 expression in femoral tissues of WT and KO mice treated with OVX or Sham surgery. **D** RT-qPCR detection and histogram of mRNA levels of P2X7 expression in femoral tissues of mice. **E** H&E staining of femoral tissue sections; **F** Representative images of mouse femur observed by micro-CT; (G) Evaluation of micro-CT parameters (Tb.Th, Tb.Sp, BV/TV, Tb.N, BMD). Bar = 100 μm and 400 μm. For (**A**, **B**), compared to the WT group, ***P < 0.001. For (**C**, **D**), compared to the WT + Sham group, ***P < 0.001. Each group consisted of six mice.

Additional file 4. Figure S4. Correlation analysis between key metabolites and key genes. **A** Heatmap showing the correlation between key differential metabolites and key genes. **B** Schematic diagram of the PI3K-AKT-GSK3β signaling pathway highlighting the key metabolites in red.

Additional file 5. Figure S5. Isolation, cultivation, and validation of mouse BMM cells. **A** Schematic diagram of the process for isolating and culturing mouse BMM cells. **B** Flow cytometry analysis of F4/80^+^ CD11b^+^ positive cells in the isolated cell population. **C** Representative images and bar graphs of TRAP staining in different groups of cells. **D** Representative images and bar graphs of the area of absorption pits detected. **E** Western Blot analysis of MMP-9, CK, and NFATc1 expression levels in cells, along with corresponding bar graphs of grayscale values. Bar = 50 μm, Mag = 150×. ***P < 0.001 compared to the Ctrl group. Cell experiments were repeated three times.

Additional file 6.

## Data Availability

All data can be provided as needed.
