# Supplementary figures and images for: New mechanistic understanding of osteoclast differentiation and bone resorption mediated by P2X7 receptors and PI3K-Akt-GSK3β signaling
Source: Cell Mol Biol Lett. 2024 Jul 8;29:100. doi: 10.1186/s11658-024-00614-5 (PMC11232284; doi:10.1186/s11658-024-00614-5)

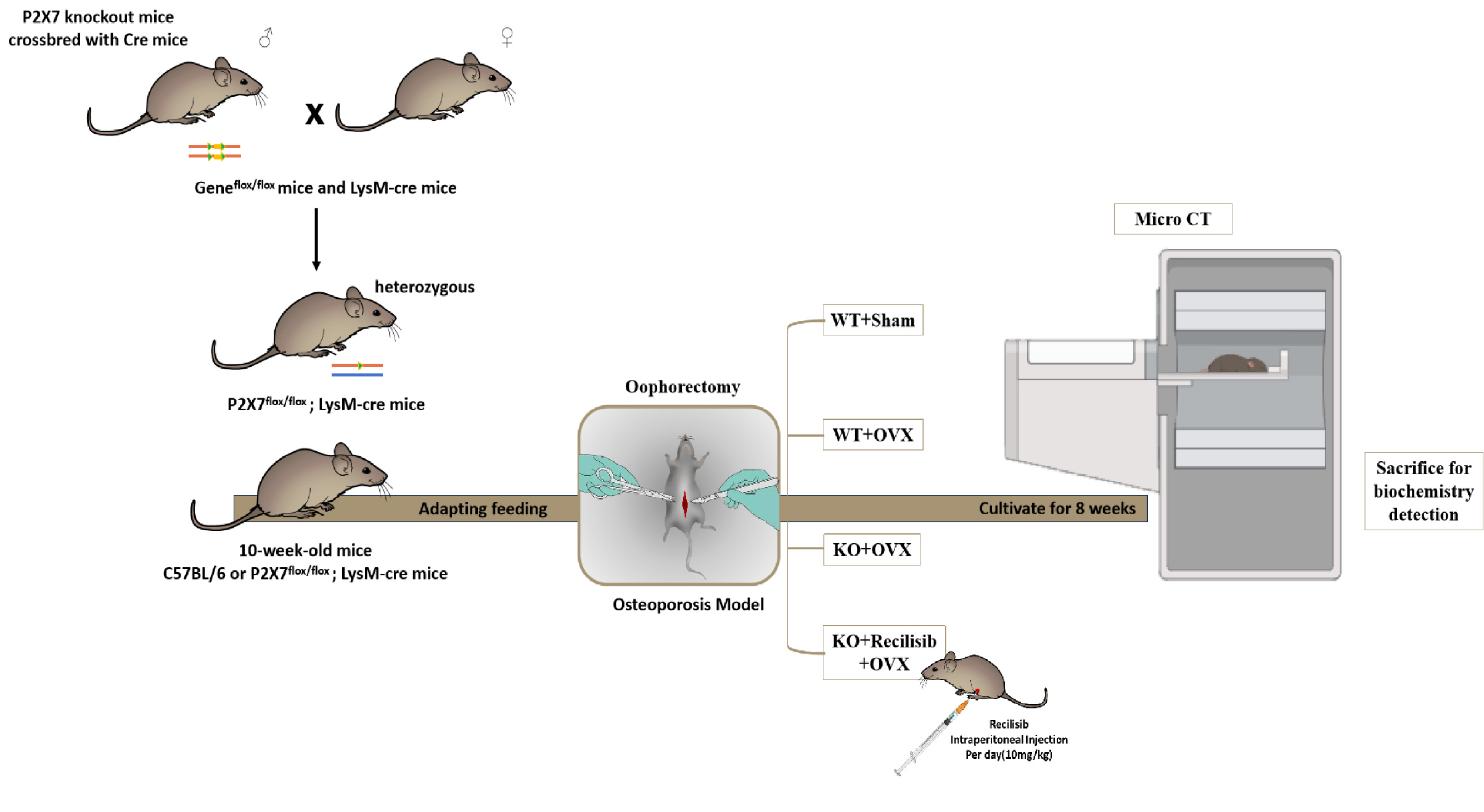

Supplement: Supplementary file 1 — Additional file 1. Figure S1. Flowchart of in vivo experimental procedure in the OP mouse model. [file 11658_2024_614_MOESM1_ESM.jpg]

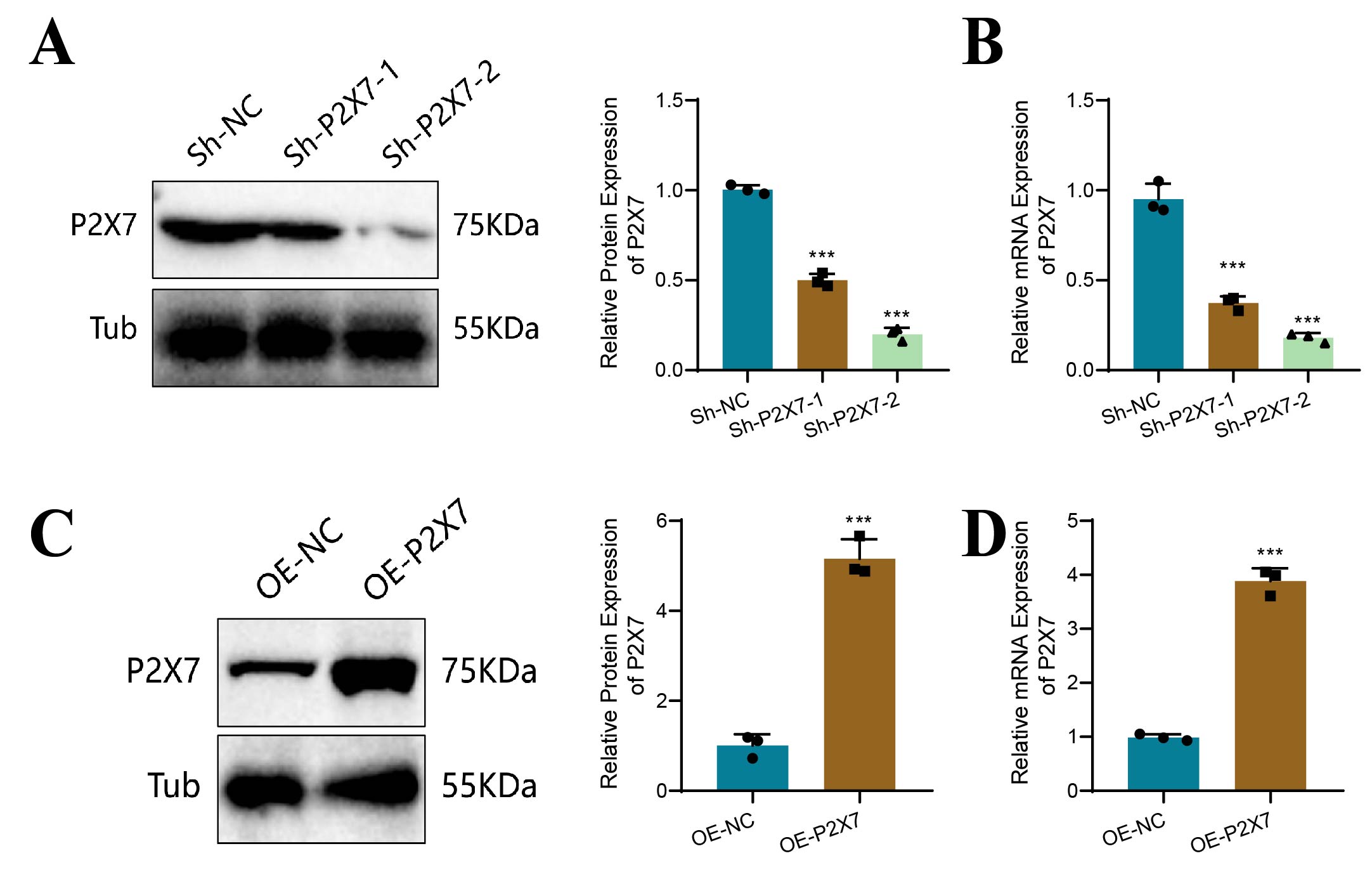

Supplement: Supplementary file 2 — Additional file 2. Figure S2. Efficiency detection of silencing lentivirus sequence and verification of lentivirus overexpression effect. A Western blot detection of P2X7 expression levels and histogram of grayscale values in each group of cells; B RT-qPCR detection of P2X7 mRNA levels in each group of cells; C Western blot detection of P2X7 expression levels and histogram of grayscale values in each group of cells; D RT-qPCR detection of P2X7 mRNA levels in each group of cells. Compared to the Sh-NC or OE-NC group, ***P < 0.001. Cell experiments were repeated three times. [file 11658_2024_614_MOESM2_ESM.jpg]

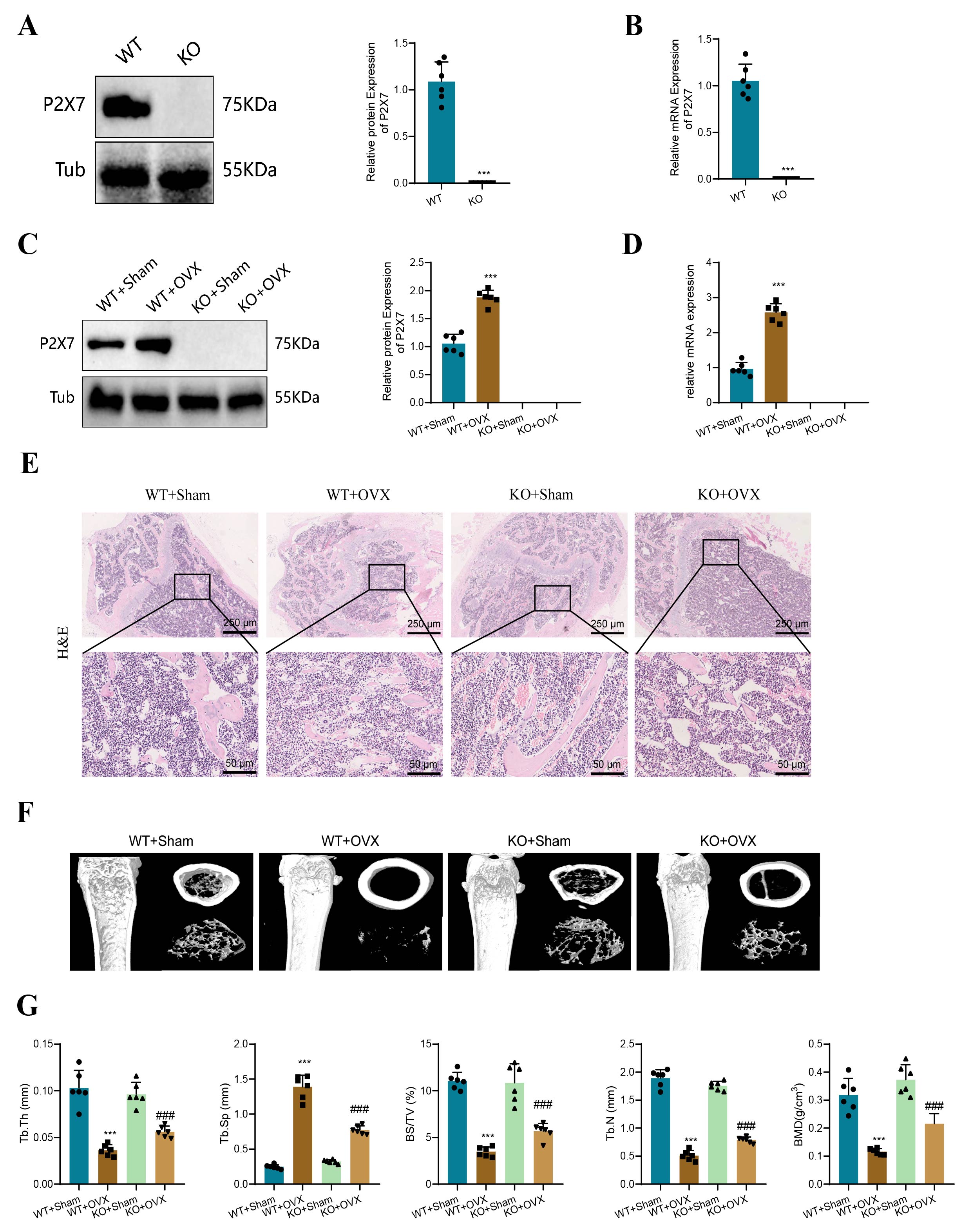

Supplement: Supplementary file 3 — Additional file 3. Figure S3. Successful validation of OP model in c57BL/6 mice and KO-P2X7 mice. A Western blot detection and histogram of grayscale values of P2X7 expression in femoral tissues of WT and KO mice. B RT-qPCR detection and histogram of mRNA levels of P2X7 expression in femoral tissues of mice. C Western blot detection and histogram of grayscale values of P2X7 expression in femoral tissues of WT and KO mice treated with OVX or Sham surgery. D RT-qPCR detection and histogram of mRNA levels of P2X7 expression in femoral tissues of mice. E H&E staining of femoral tissue sections; F Representative images of mouse femur observed by micro-CT; (G) Evaluation of micro-CT parameters (Tb.Th, Tb.Sp, BV/TV, Tb.N, BMD). Bar = 100 μm and 400 μm. For (A, B), compared to the WT group, ***P < 0.001. For (C, D), compared to the WT + Sham group, ***P < 0.001. Each group consisted of six mice. [file 11658_2024_614_MOESM3_ESM.jpg]

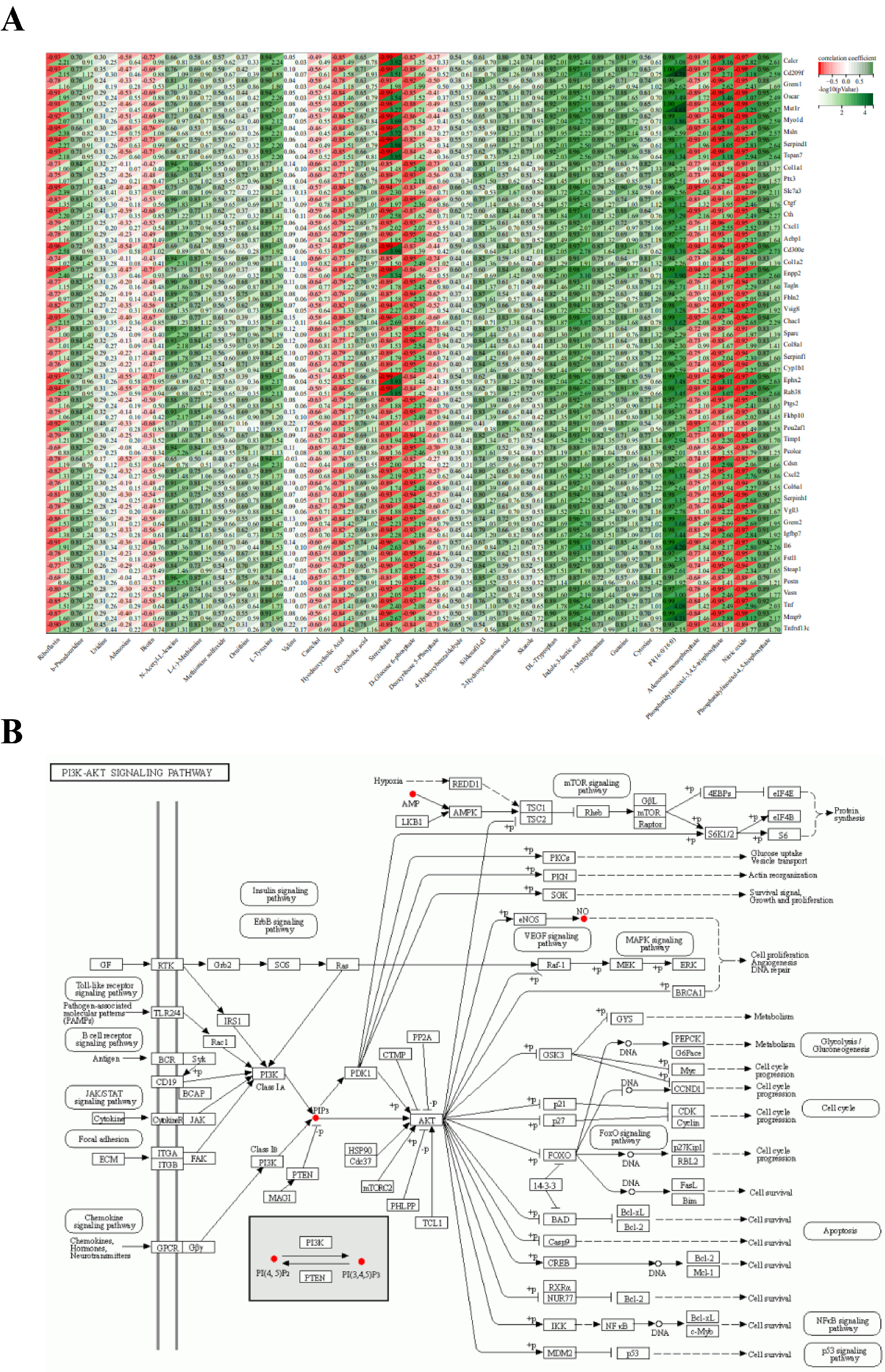

Supplement: Supplementary file 4 — Additional file 4. Figure S4. Correlation analysis between key metabolites and key genes. A Heatmap showing the correlation between key differential metabolites and key genes. B Schematic diagram of the PI3K-AKT-GSK3β signaling pathway highlighting the key metabolites in red. [file 11658_2024_614_MOESM4_ESM.jpg]

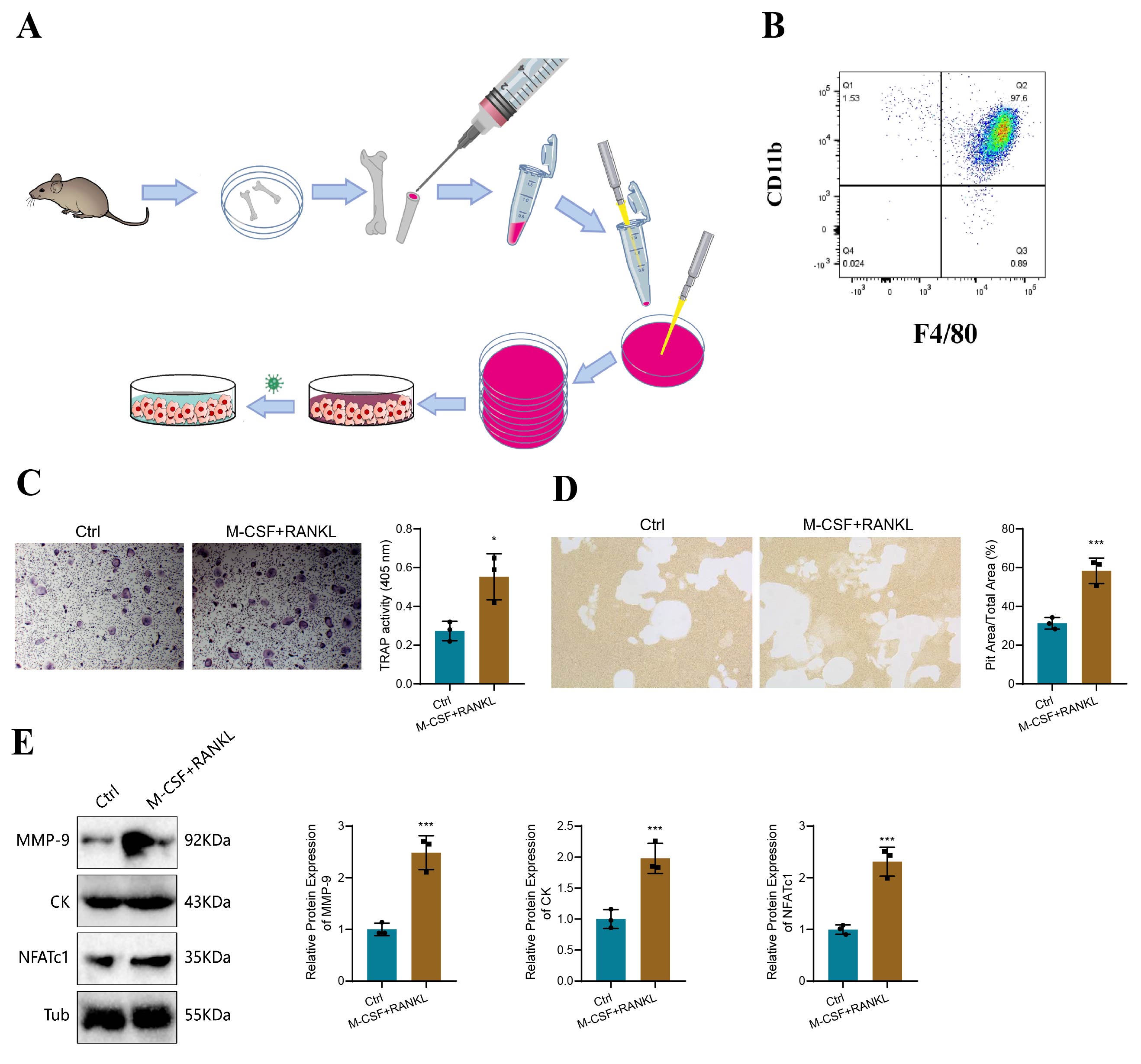

Supplement: Supplementary file 5 — Additional file 5. Figure S5. Isolation, cultivation, and validation of mouse BMM cells. A Schematic diagram of the process for isolating and culturing mouse BMM cells. B Flow cytometry analysis of F4/80+ CD11b+ positive cells in the isolated cell population. C Representative images and bar graphs of TRAP staining in different groups of cells. D Representative images and bar graphs of the area of absorption pits detected. E Western Blot analysis of MMP-9, CK, and NFATc1 expression levels in cells, along with corresponding bar graphs of grayscale values. Bar = 50 μm, Mag = 150×. ***P < 0.001 compared to the Ctrl group. Cell experiments were repeated three times. [file 11658_2024_614_MOESM5_ESM.jpg]
