# Supplementary material for: New mechanistic understanding of osteoclast differentiation and bone resorption mediated by P2X7 receptors and PI3K-Akt-GSK3β signaling
Source: Cell Mol Biol Lett. 2024 Jul 8;29:100. doi: 10.1186/s11658-024-00614-5 (PMC11232284; doi:10.1186/s11658-024-00614-5)
Supplement: Supplementary file 6 — Additional file 6. [file 11658_2024_614_MOESM6_ESM.docx]

**Table S1. Silencing P2X7 lentivirus sequence**

| **Gene** | **Sequences** |
| --- | --- |
| Sh-NC | 5'-CCTAAGGTTAAGTCGCCCTCG-3' |
| Sh-P2X7-1 | 5'- GCATGAATTATGGCACCATTA-3' |
| Sh-P2X7-2 | 5'- CCGAGAAACAGGCGATAATTT-3' |

**Table S2. RT-qPCR primer sequence**

| **Gene** | **Sequences** |
| --- | --- |
| P2rx7(Mouse) | Forward: 5'-GCCAAGAAGTTCCAACCTAGATAC-3'  Reverse: 5'-TCCATTGAGAGCATGGCTTCTTG-3' |
| Gapdh(Mouse) | Forward: 5'- AGGTCGGTGTGAACGGATTTG -3' |
|  | Reverse: 5'- GGGGTCGTTGATGGCAACA -3' |

**Table S3. 540 differentially expressed genes**

| **Gene** | **LogFC** | **P.Value** |
| --- | --- | --- |
| Cth | 5.347670522 | 1.98E-12 |
| Il6 | 4.167214569 | 1.99E-12 |
| Cd209f | 6.403207325 | 3.48E-12 |
| Enpp2 | 4.838217443 | 4.51E-12 |
| Myo1d | 5.804439965 | 6.23E-12 |
| Dmkn | -3.817294489 | 6.37E-12 |
| Grem2 | 4.205285674 | 7.62E-12 |
| Zfp811 | 3.631609795 | 8.14E-12 |
| Calcr | 6.699975854 | 1.19E-11 |
| Edn1 | 3.697454813 | 1.25E-11 |
| Retnlg | -3.540608414 | 1.52E-11 |
| Mst1r | 5.853581587 | 1.88E-11 |
| Ppap2a | 3.872372601 | 2.05E-11 |
| Nfkbiz | 3.160803147 | 2.16E-11 |
| Oscar | 5.861968264 | 2.46E-11 |
| Tspan7 | 5.698690145 | 2.60E-11 |
| Socs3 | 3.144551051 | 3.26E-11 |
| Cd300e | 4.930818784 | 3.43E-11 |
| Gm13571 | 2.693207366 | 5.56E-11 |
| Il1a | 3.732876709 | 6.03E-11 |
| Prss23 | 2.662578277 | 6.04E-11 |
| Tnf | 4.088137528 | 6.10E-11 |
| Adamts14 | 3.709223877 | 6.40E-11 |
| Serpinb2 | 3.456573556 | 8.40E-11 |
| Ccl3 | 2.661731796 | 8.48E-11 |
| Chac1 | 4.716308675 | 1.05E-10 |
| Slc7a3 | 5.512466109 | 1.07E-10 |
| Ccl4 | 3.549125243 | 1.10E-10 |
| Ephx2 | 4.542730953 | 1.17E-10 |
| Ckb | 3.260302095 | 1.18E-10 |
| Lrrc32 | 3.171195781 | 1.40E-10 |
| Fam109a | 2.799249065 | 1.42E-10 |
| St8sia6 | 3.364624724 | 1.51E-10 |
| Fbxl16 | 3.670854697 | 1.62E-10 |
| Gipr | 2.532108732 | 1.67E-10 |
| Vsig8 | 4.764788505 | 1.80E-10 |
| Snai2 | 3.984138662 | 1.85E-10 |
| Plekhs1 | -2.450768229 | 2.03E-10 |
| Hmga2-ps1 | -2.706381375 | 2.03E-10 |
| Cd55 | 3.080283864 | 2.08E-10 |
| Slc13a3 | 2.50903074 | 2.22E-10 |
| Ctsk | 3.143080367 | 2.29E-10 |
| Rab38 | 4.537493668 | 2.56E-10 |
| Mmp9 | 4.081417773 | 2.71E-10 |
| Cd209g | 2.972757133 | 2.89E-10 |
| F3 | 3.089386903 | 2.96E-10 |
| Lox | 2.951738271 | 2.97E-10 |
| Urah | 3.689049846 | 3.15E-10 |
| Gdf15 | 2.373297566 | 3.17E-10 |
| Hbegf | 3.106032253 | 3.35E-10 |
| Ier5l | 2.776716952 | 3.40E-10 |
| Cpe | 3.373845164 | 3.66E-10 |
| Adamts2 | 3.94298994 | 3.71E-10 |
| Cxcl2 | 4.28911147 | 3.73E-10 |
| Steap4 | 3.520873201 | 3.73E-10 |
| Hist2h3c2 | 3.197152088 | 3.84E-10 |
| Ctgf | 5.401781093 | 4.24E-10 |
| Marco | 2.930664412 | 4.35E-10 |
| Slc9b2 | 3.854478924 | 4.52E-10 |
| Foxf2 | 2.724095822 | 4.56E-10 |
| Serpind1 | 5.702639643 | 4.66E-10 |
| Dntt | -2.182431451 | 4.85E-10 |
| Col1a1 | 5.598758865 | 5.06E-10 |
| Cxcl1 | 5.127485353 | 5.13E-10 |
| Epb4.1l3 | 2.880818855 | 5.29E-10 |
| Timp1 | 4.348171809 | 5.34E-10 |
| Ptx3 | 5.560683212 | 5.38E-10 |
| Wisp1 | 4.03813626 | 5.38E-10 |
| Card14 | 2.833389878 | 5.42E-10 |
| Aldh1l2 | 3.158698741 | 5.44E-10 |
| Serpinf1 | 4.591659106 | 5.84E-10 |
| Hcar2 | 2.66514649 | 6.00E-10 |
| Inhbb | 3.778067431 | 6.19E-10 |
| Tagln | 4.812509357 | 6.34E-10 |
| Vasn | 4.090787131 | 6.56E-10 |
| C77080 | 2.169437692 | 6.64E-10 |
| Pim2 | 3.625350794 | 6.80E-10 |
| Fkbp10 | 4.363322023 | 7.01E-10 |
| Dppa3 | -2.069896436 | 7.34E-10 |
| Ddit4 | 2.651570234 | 7.44E-10 |
| Vgll3 | 4.247620664 | 7.70E-10 |
| Tmem98 | 2.043019509 | 7.91E-10 |
| Nhs | 2.059021042 | 7.95E-10 |
| Apbb1 | 2.563630938 | 8.27E-10 |
| Fstl1 | 4.158035412 | 9.12E-10 |
| Ldhb | 3.411071095 | 9.28E-10 |
| Msi2 | 2.628882935 | 9.34E-10 |
| Elovl6 | -2.134537404 | 9.65E-10 |
| Ppm1n | 2.724403132 | 1.00E-09 |
| Il10 | 3.162122594 | 1.02E-09 |
| Fkbp9 | 3.005142119 | 1.05E-09 |
| Aebp1 | 5.063304273 | 1.08E-09 |
| Col1a2 | 4.930688351 | 1.10E-09 |
| Ppic | 3.262363554 | 1.10E-09 |
| 1700003M07Rik | 3.700366736 | 1.11E-09 |
| Daf2 | 2.592173262 | 1.11E-09 |
| Rufy4 | 2.965550659 | 1.12E-09 |
| Prdm8 | 3.066245528 | 1.23E-09 |
| Car2 | 3.237064631 | 1.24E-09 |
| Cited1 | 2.176457732 | 1.25E-09 |
| Trib3 | 3.352914067 | 1.27E-09 |
| Serpine2 | 2.380062784 | 1.31E-09 |
| Fam64a | -2.429110046 | 1.32E-09 |
| Fjx1 | 2.327712256 | 1.32E-09 |
| Cdc42ep1 | 3.133172195 | 1.33E-09 |
| D13Ertd608e | -2.189824737 | 1.38E-09 |
| Irg1 | 2.16497141 | 1.41E-09 |
| Lum | 3.298385843 | 1.41E-09 |
| Ryk | 2.455353799 | 1.42E-09 |
| Cd200 | 3.330206849 | 1.45E-09 |
| Ppfia3 | 3.455399359 | 1.45E-09 |
| Krt7 | 2.528317337 | 1.48E-09 |
| Stac2 | 2.964770979 | 1.48E-09 |
| Ppm1j | 2.044698913 | 1.50E-09 |
| Batf | 3.052122451 | 1.56E-09 |
| Svip | -2.039106301 | 1.63E-09 |
| Col6a3 | 3.264554074 | 1.64E-09 |
| Tmem178 | 3.570777267 | 1.65E-09 |
| 3930401B19Rik | 2.049871011 | 1.73E-09 |
| Kbtbd11 | 3.681212338 | 1.74E-09 |
| Mfge8 | 2.454338986 | 1.76E-09 |
| Epha4 | 2.445345711 | 1.85E-09 |
| Pkp2 | 3.201578561 | 2.00E-09 |
| Shisa3 | 3.676664134 | 2.03E-09 |
| Pxdn | 2.341242701 | 2.20E-09 |
| Akr1c18 | 3.510391059 | 2.30E-09 |
| Csf3 | 3.150421443 | 2.40E-09 |
| Zfp827 | 2.164645529 | 2.50E-09 |
| Col4a5 | -2.102118253 | 2.52E-09 |
| 4833415N18Rik | 3.574344718 | 2.55E-09 |
| Wisp2 | 3.227517471 | 2.56E-09 |
| Grem1 | 5.909849871 | 2.59E-09 |
| Dab2 | -2.279757278 | 2.61E-09 |
| Msln | 5.782435803 | 2.70E-09 |
| Nr4a1 | 3.324641943 | 2.73E-09 |
| Klrb1a | 2.206395329 | 2.79E-09 |
| Prss57 | -2.369316635 | 2.79E-09 |
| Osmr | 3.410201502 | 2.88E-09 |
| Il12a | 2.010166237 | 2.89E-09 |
| Skint3 | -2.090135467 | 2.90E-09 |
| Fbln2 | 4.786056734 | 2.93E-09 |
| Fpr1 | 2.825979576 | 2.96E-09 |
| Ccbe1 | 2.226741662 | 2.97E-09 |
| Loxl1 | 4.276770682 | 3.01E-09 |
| Gm19705 | 2.124686579 | 3.14E-09 |
| Epn2 | 2.133863381 | 3.16E-09 |
| Cck | 3.939616671 | 3.24E-09 |
| Tpm2 | 2.860982352 | 3.29E-09 |
| Tbx3 | 2.931397862 | 3.33E-09 |
| Bdh1 | 2.645980595 | 3.33E-09 |
| Tnfrsf11b | 2.275854768 | 3.34E-09 |
| Cyr61 | 3.859440982 | 3.35E-09 |
| Fat1 | 3.295700467 | 3.42E-09 |
| Slc31a2 | 2.321890913 | 3.49E-09 |
| Siglec15 | 3.730441111 | 3.50E-09 |
| Sparc | 4.710064062 | 3.54E-09 |
| Alpl | 3.159029045 | 3.60E-09 |
| Cd38 | 2.804807955 | 3.62E-09 |
| Maff | 2.787392003 | 3.75E-09 |
| Prg3 | -2.134655387 | 3.76E-09 |
| Atp6v0d2 | 3.116518964 | 3.86E-09 |
| Vldlr | 2.850636158 | 3.92E-09 |
| Lpar6 | -2.071866596 | 3.97E-09 |
| St5 | 2.094869851 | 4.13E-09 |
| Bcat1 | 4.030956722 | 4.14E-09 |
| Ext1 | 2.074272437 | 4.32E-09 |
| Lancl3 | 2.104510691 | 4.43E-09 |
| Arap2 | 2.468808582 | 4.44E-09 |
| Serpinh1 | 4.265295486 | 4.56E-09 |
| Hspb1 | 3.558319213 | 4.56E-09 |
| Arsb | 2.324096358 | 4.65E-09 |
| Gpr176 | 3.291163558 | 4.79E-09 |
| Sez6l2 | 2.241928928 | 4.79E-09 |
| Fhl2 | 3.29293219 | 4.82E-09 |
| Bmp1 | 2.868510494 | 4.91E-09 |
| Gm4610 | 3.143842033 | 4.96E-09 |
| Slc32a1 | 2.310890272 | 5.00E-09 |
| Igfbp7 | 4.18824665 | 5.09E-09 |
| Serinc2 | 3.618093993 | 5.27E-09 |
| Dtx1 | 3.996734989 | 5.40E-09 |
| Myl10 | -2.192053043 | 5.45E-09 |
| Cxcr2 | -2.04607379 | 5.46E-09 |
| Arhgef39 | -2.571768527 | 5.52E-09 |
| Cst6 | 2.011882427 | 5.70E-09 |
| Anln | -2.382942436 | 5.83E-09 |
| Fermt2 | 3.680852144 | 5.86E-09 |
| Gm6756 | 2.443438273 | 5.87E-09 |
| Raver2 | 3.798260106 | 5.89E-09 |
| Nid1 | 2.79016473 | 5.91E-09 |
| Spns2 | 3.666377248 | 5.95E-09 |
| Ccr6 | 3.004784148 | 5.96E-09 |
| Prrx1 | 2.209001555 | 6.02E-09 |
| Steap1 | 4.14251439 | 6.04E-09 |
| Pip5k1b | 2.449767446 | 6.14E-09 |
| Cd248 | 3.351413068 | 6.22E-09 |
| Stc2 | 2.999862353 | 6.32E-09 |
| Kank1 | 2.38057506 | 6.37E-09 |
| Edil3 | 2.936567958 | 6.49E-09 |
| Tnfrsf13c | 4.059126856 | 6.52E-09 |
| Irak3 | 2.210102439 | 6.67E-09 |
| Spry1 | 2.744955025 | 6.67E-09 |
| Crim1 | 2.831907403 | 6.73E-09 |
| 2210008F06Rik | 2.570927121 | 6.95E-09 |
| B930041F14Rik | 2.037999394 | 7.22E-09 |
| Sphk1 | 3.167864851 | 7.43E-09 |
| Ascl2 | 3.628040962 | 7.50E-09 |
| Ms4a3 | -3.227314087 | 7.67E-09 |
| Clec4e | 2.132699184 | 7.86E-09 |
| Cyp1b1 | 4.563046979 | 7.99E-09 |
| Rapgef5 | 2.362264621 | 8.30E-09 |
| Cx3cr1 | -4.451199906 | 8.32E-09 |
| Plekhg5 | 2.08021673 | 8.41E-09 |
| Cp | 2.584707567 | 8.42E-09 |
| Ch25h | 3.14453879 | 8.52E-09 |
| Tnfrsf25 | 2.086488583 | 8.71E-09 |
| Col8a1 | 4.614189617 | 8.73E-09 |
| Trim54 | 2.380544205 | 8.84E-09 |
| Prkcdbp | 2.162583371 | 8.87E-09 |
| Mras | 2.344166169 | 8.93E-09 |
| Adm | 3.328273212 | 9.27E-09 |
| Cnn3 | 2.614614863 | 9.69E-09 |
| Wnt10a | 3.813207621 | 9.73E-09 |
| Gpr31b | 3.057003994 | 9.82E-09 |
| Col6a1 | 4.288537979 | 9.82E-09 |
| Bcl2l14 | -2.052033105 | 9.97E-09 |
| Crlf1 | 2.258549874 | 1.00E-08 |
| Col3a1 | 3.127714842 | 1.05E-08 |
| Col4a1 | 2.734146167 | 1.08E-08 |
| Heg1 | 2.249742912 | 1.09E-08 |
| Itga9 | 2.097774639 | 1.10E-08 |
| Postn | 4.127950277 | 1.12E-08 |
| Enah | 2.374172247 | 1.12E-08 |
| Gstt1 | -2.140958734 | 1.13E-08 |
| Gm15987 | 3.071011586 | 1.14E-08 |
| Lsr | -2.762196962 | 1.15E-08 |
| Adgrg6 | 2.247721712 | 1.16E-08 |
| Fgf7 | 3.652232115 | 1.17E-08 |
| Syde1 | 2.915870444 | 1.25E-08 |
| Fam102a | 2.17803228 | 1.26E-08 |
| Gm16049 | -2.074368797 | 1.27E-08 |
| Ramp3 | -2.462660971 | 1.27E-08 |
| Inhba | 3.54193067 | 1.32E-08 |
| Tfrc | 2.281343018 | 1.36E-08 |
| Serpine1 | 3.335501416 | 1.36E-08 |
| Hist2h3c1 | 2.010993542 | 1.37E-08 |
| Gimap6 | 2.733490473 | 1.46E-08 |
| Jam3 | 2.475411841 | 1.47E-08 |
| Susd3 | -2.092600784 | 1.47E-08 |
| Tgfb2 | 2.217039536 | 1.47E-08 |
| Pcolce | 4.326158187 | 1.50E-08 |
| Pmepa1 | 2.335728046 | 1.50E-08 |
| Ptgs2 | 4.407904504 | 1.53E-08 |
| Id3 | 2.242878534 | 1.54E-08 |
| Gm5086 | -2.095972756 | 1.55E-08 |
| Hrh3 | 2.501997639 | 1.63E-08 |
| Rnf150 | -2.072860452 | 1.63E-08 |
| Clmp | 2.699094256 | 1.65E-08 |
| Hic1 | 2.982612001 | 1.66E-08 |
| Cd28 | -2.464895041 | 1.69E-08 |
| Lamb1 | 3.208132967 | 1.74E-08 |
| Gpr18 | 2.424085375 | 1.77E-08 |
| Cdh2 | 2.088908118 | 1.77E-08 |
| Fads3 | 2.676578459 | 1.77E-08 |
| Psph | 2.046250445 | 1.82E-08 |
| Twist2 | 2.274575757 | 1.83E-08 |
| Epdr1 | 2.381520359 | 1.83E-08 |
| Col5a3 | 3.17868511 | 1.83E-08 |
| Lhfp | 2.188879764 | 1.84E-08 |
| Emx2 | 2.516586457 | 1.84E-08 |
| Kirrel | 2.267874091 | 1.92E-08 |
| Adamts4 | 2.717192689 | 1.96E-08 |
| Gm13546 | -2.364949551 | 1.99E-08 |
| Bdh2 | 2.479024178 | 2.00E-08 |
| Col16a1 | 2.428500599 | 2.01E-08 |
| Ankrd2 | 2.216655239 | 2.02E-08 |
| Fn1 | 3.519188602 | 2.07E-08 |
| Folr2 | -2.898415839 | 2.19E-08 |
| Gm15674 | 2.221470444 | 2.21E-08 |
| Cd101 | -2.068727125 | 2.23E-08 |
| Fbln7 | 2.423050817 | 2.28E-08 |
| Dlk1 | 3.10220636 | 2.34E-08 |
| Ebf1 | 2.816758349 | 2.45E-08 |
| Bcl2a1c | 2.3301054 | 2.49E-08 |
| Ccr5 | -2.076422961 | 2.53E-08 |
| Ccr2 | -3.284720133 | 2.54E-08 |
| Cnksr3 | 2.491013638 | 2.56E-08 |
| Sod2 | 2.296472112 | 2.60E-08 |
| Rnf144b | -2.095073554 | 2.60E-08 |
| Ccrl2 | 2.00789186 | 2.68E-08 |
| Bcl6b | 2.193836482 | 2.72E-08 |
| Fbln5 | 2.867598421 | 2.76E-08 |
| Mmp2 | 3.330214384 | 2.89E-08 |
| Bex6 | -2.24783138 | 2.90E-08 |
| Gimap5 | 3.62624771 | 2.90E-08 |
| Adra2a | 2.211037354 | 2.97E-08 |
| Ppp3cc | 2.126098906 | 3.02E-08 |
| Gm6377 | 2.060878991 | 3.08E-08 |
| Slc6a4 | -2.363337687 | 3.11E-08 |
| Foxc2 | 2.37935364 | 3.13E-08 |
| Mfsd7c | 2.398300853 | 3.20E-08 |
| Klf15 | 2.090448893 | 3.21E-08 |
| Gm32819 | 2.592146355 | 3.34E-08 |
| Clec10a | -3.265084681 | 3.35E-08 |
| Cenpf | -2.699405464 | 3.39E-08 |
| Adgra3 | 3.040454791 | 3.46E-08 |
| Pou2af1 | 4.34997663 | 3.48E-08 |
| Cd109 | 2.097757292 | 3.53E-08 |
| Asns | 2.365795481 | 3.53E-08 |
| Mmp14 | 2.085586817 | 3.61E-08 |
| Gm8096 | 2.513307663 | 3.66E-08 |
| Gem | 2.776537649 | 3.70E-08 |
| Tlr5 | -2.148665141 | 3.72E-08 |
| Dpysl3 | 2.613080907 | 3.87E-08 |
| Dusp4 | 2.126001071 | 3.91E-08 |
| Cdsn | 4.296791379 | 3.94E-08 |
| Pcdh19 | 2.304135119 | 3.95E-08 |
| Unc5b | 3.16695189 | 4.09E-08 |
| Fabp3 | 2.45735885 | 4.46E-08 |
| Cd19 | 3.596342673 | 4.52E-08 |
| Ccl20 | 3.336692308 | 4.57E-08 |
| Cdr2l | 2.509636915 | 4.58E-08 |
| Tnc | 2.381571966 | 4.72E-08 |
| Itgb3 | 2.093178052 | 4.90E-08 |
| Traf4 | 2.995636448 | 5.05E-08 |
| Lta | 2.174677289 | 5.09E-08 |
| Ddr2 | 2.362038206 | 5.29E-08 |
| Evpl | 2.339807569 | 5.33E-08 |
| Dcbld2 | 2.202092685 | 5.38E-08 |
| Mboat1 | -2.021117456 | 5.49E-08 |
| Dcbld1 | 3.025032177 | 5.55E-08 |
| Rspo2 | 2.773137874 | 5.66E-08 |
| Csf1 | 3.39827883 | 5.68E-08 |
| Cdh17 | 2.603910806 | 5.72E-08 |
| Elane | -3.280755837 | 5.82E-08 |
| Acoxl | 2.449849455 | 5.89E-08 |
| Farp1 | 2.230084171 | 5.97E-08 |
| Cald1 | 2.288681936 | 6.33E-08 |
| Egr1 | 2.947474052 | 6.44E-08 |
| Met | 2.00279588 | 6.55E-08 |
| Corin | 2.301267677 | 6.80E-08 |
| Samd4 | 2.006701778 | 6.88E-08 |
| Prl2c1 | 2.104638592 | 7.35E-08 |
| BC021767 | -2.106430962 | 7.41E-08 |
| Phgdh | 2.458160477 | 7.41E-08 |
| Pitx1 | 2.204613219 | 7.54E-08 |
| Egr3 | 2.514536651 | 7.78E-08 |
| Apol7c | -2.097680786 | 7.83E-08 |
| Kif23 | -2.096095034 | 8.07E-08 |
| Igf2bp2 | 2.001638818 | 8.46E-08 |
| Sdc2 | 3.213600294 | 8.51E-08 |
| Satb1 | 2.382400791 | 8.61E-08 |
| Plac9a | 3.225223175 | 8.79E-08 |
| Lppr3 | -2.40319967 | 8.85E-08 |
| Kif20a | -2.373980216 | 8.86E-08 |
| Arhgef25 | 2.311281836 | 9.14E-08 |
| Pcbd1 | 3.224083668 | 9.27E-08 |
| Amigo2 | 3.487119264 | 9.35E-08 |
| Lurap1l | 3.439349857 | 9.43E-08 |
| Ets2 | 2.15604116 | 9.46E-08 |
| Pdcd1 | 3.281077328 | 9.75E-08 |
| Map1b | 2.52288888 | 9.79E-08 |
| Tg | 2.059118611 | 9.82E-08 |
| Tm4sf1 | 3.066402582 | 1.01E-07 |
| Mzb1 | 3.795719543 | 1.02E-07 |
| Phldb2 | 2.181694687 | 1.04E-07 |
| Crip2 | 2.593706341 | 1.07E-07 |
| Cdkn3 | -2.007346829 | 1.09E-07 |
| Pdgfrb | 3.125773103 | 1.10E-07 |
| Cyp26b1 | 2.435216055 | 1.12E-07 |
| Dlg5 | 2.079468187 | 1.15E-07 |
| Kctd15 | 2.372813542 | 1.21E-07 |
| Gm34084 | -2.464582582 | 1.21E-07 |
| Shisa8 | 3.703748607 | 1.24E-07 |
| Rcn3 | 2.494267752 | 1.26E-07 |
| Ankle1 | -2.197286134 | 1.27E-07 |
| Ifitm1 | -2.957106676 | 1.29E-07 |
| Fbn1 | 2.394979494 | 1.37E-07 |
| Nbl1 | 2.263119395 | 1.39E-07 |
| Gm15056 | -2.166735116 | 1.40E-07 |
| Tmeff1 | 2.033132624 | 1.40E-07 |
| Ccnb2 | -2.321082192 | 1.42E-07 |
| Gpx8 | 2.828265811 | 1.43E-07 |
| Mir99ahg | -2.43248759 | 1.46E-07 |
| Slc35g1 | 2.01227287 | 1.48E-07 |
| Col6a2 | 2.825013119 | 1.53E-07 |
| Tmem45a | 2.925472682 | 1.58E-07 |
| Arhgef17 | 2.709049523 | 1.61E-07 |
| Mef2b | 3.639657533 | 1.64E-07 |
| Hnf1b | 3.327958293 | 1.64E-07 |
| Cyb561 | 2.255936494 | 1.66E-07 |
| Dcn | 2.317320275 | 1.67E-07 |
| U90926 | 2.710407571 | 1.68E-07 |
| Plk1 | -2.528124661 | 1.72E-07 |
| Tmem204 | 2.291981203 | 1.74E-07 |
| Ctsg | -3.417902391 | 1.74E-07 |
| Perp | 2.073328864 | 1.79E-07 |
| Mpo | -3.426838262 | 1.79E-07 |
| Rgs16 | 3.345375435 | 1.82E-07 |
| 9130008F23Rik | -2.066201441 | 1.85E-07 |
| Nyap2 | 2.253917939 | 1.88E-07 |
| F13a1 | -2.943632998 | 1.96E-07 |
| Myo1b | 2.523979429 | 1.97E-07 |
| Rasgrp3 | 2.525936635 | 1.97E-07 |
| Mark1 | 2.041076867 | 1.97E-07 |
| Ckap2 | -2.163092182 | 1.98E-07 |
| Bicc1 | 3.010927392 | 2.09E-07 |
| LOC102634683 | 2.127332242 | 2.13E-07 |
| Slc6a9 | 2.399095679 | 2.14E-07 |
| Ndrg1 | 2.069594546 | 2.17E-07 |
| Scd1 | 2.781590211 | 2.21E-07 |
| Epha2 | 2.240666289 | 2.21E-07 |
| Dusp14 | 2.436998519 | 2.29E-07 |
| Nsun7 | 2.372903512 | 2.35E-07 |
| Mgp | 2.991825448 | 2.36E-07 |
| Slc35f2 | 3.108580861 | 2.51E-07 |
| Sema3b | 3.386162129 | 2.53E-07 |
| Itgax | 2.344780323 | 2.53E-07 |
| Cdca3 | -2.013832193 | 2.54E-07 |
| Wnt10b | 2.367657425 | 2.65E-07 |
| A430093F15Rik | 2.479090704 | 2.65E-07 |
| Dpep1 | 2.510745001 | 2.66E-07 |
| Nnmt | 2.548810323 | 2.67E-07 |
| Gimap1 | 3.087659623 | 2.91E-07 |
| Gimap4 | 2.461257424 | 2.94E-07 |
| Cysrt1 | -2.77007143 | 2.97E-07 |
| Wwtr1 | 2.845338843 | 3.02E-07 |
| Cd83 | 2.461134113 | 3.18E-07 |
| Fah | 2.02688128 | 3.22E-07 |
| Cxcl3 | 2.139035087 | 3.24E-07 |
| Rab30 | 2.515687483 | 3.34E-07 |
| Dpp4 | 2.333260969 | 3.39E-07 |
| Ctla4 | 3.609607741 | 3.41E-07 |
| Avpr1a | 2.510754352 | 3.50E-07 |
| Ifitm6 | -2.173997843 | 3.70E-07 |
| Thbs2 | 3.356759944 | 3.71E-07 |
| Dkk3 | 2.950046354 | 3.84E-07 |
| P3h4 | 2.428379476 | 3.84E-07 |
| Aqp1 | 2.899714211 | 3.85E-07 |
| Cdkn2b | 2.34620949 | 4.00E-07 |
| Prtn3 | -3.287649988 | 4.08E-07 |
| Gimap3 | 2.715272965 | 4.25E-07 |
| Sod3 | 3.398630534 | 4.26E-07 |
| Fam46c | 2.188807915 | 4.32E-07 |
| Gm11545 | -2.192029544 | 4.35E-07 |
| Aspm | -2.567865732 | 4.41E-07 |
| Ablim1 | 2.029097382 | 4.44E-07 |
| Ptprcap | 2.87115647 | 4.62E-07 |
| Cxcr5 | 2.900099858 | 4.65E-07 |
| Fgf21 | 2.19042218 | 4.65E-07 |
| Acpp | 2.009072709 | 4.78E-07 |
| Irf4 | 2.932850168 | 4.82E-07 |
| Lck | 2.507259433 | 5.00E-07 |
| Cd69 | 2.528628028 | 5.00E-07 |
| Trib2 | 2.170306134 | 5.08E-07 |
| Ccna2 | -2.038049984 | 5.18E-07 |
| Ppp1r16b | 2.353990526 | 5.26E-07 |
| Cpm | 2.182002306 | 5.59E-07 |
| 1810010D01Rik | -2.928420148 | 5.60E-07 |
| Tbx21 | 2.625112975 | 5.77E-07 |
| Pipox | 2.609345156 | 5.80E-07 |
| Loxl2 | 2.55960398 | 5.82E-07 |
| Col4a2 | 2.252009483 | 6.22E-07 |
| Soat2 | 2.252140802 | 6.34E-07 |
| Grhl1 | 2.328687739 | 6.45E-07 |
| Slfn4 | -2.646961426 | 6.66E-07 |
| Fcrla | 3.066593361 | 6.96E-07 |
| Aicda | 3.611403852 | 6.98E-07 |
| Siglec1 | -2.026159083 | 7.43E-07 |
| Ms4a1 | 2.76746965 | 7.67E-07 |
| Cdc20 | -2.047139881 | 7.72E-07 |
| Ikzf3 | 2.365897117 | 7.91E-07 |
| Eml1 | 2.150147382 | 8.30E-07 |
| Nsg1 | 2.324205266 | 8.59E-07 |
| Als2cr12 | -2.01198597 | 9.75E-07 |
| Bcas1 | -2.647491225 | 1.03E-06 |
| Abcg3 | -2.301194487 | 1.05E-06 |
| Ltbp2 | 2.09342603 | 1.10E-06 |
| P4ha2 | 2.147602293 | 1.15E-06 |
| Pgf | -2.350725265 | 1.16E-06 |
| Gpr171 | 2.52426984 | 1.21E-06 |
| 2010005H15Rik | -2.216343035 | 1.24E-06 |
| Cxcl13 | 3.63431461 | 1.28E-06 |
| Itga11 | 2.701763483 | 1.28E-06 |
| Timp3 | 2.942405382 | 1.30E-06 |
| Fam20a | 2.773152312 | 1.33E-06 |
| Cxcl5 | 3.05733141 | 1.43E-06 |
| Ly6d | 2.735543316 | 1.43E-06 |
| Irx3 | 2.050850009 | 1.48E-06 |
| Ccdc80 | 2.706056474 | 1.53E-06 |
| Il1r1 | 2.000496041 | 1.62E-06 |
| Gzmc | 3.580300932 | 1.71E-06 |
| Ifng | 2.614552213 | 1.76E-06 |
| Timd2 | 3.054252131 | 1.83E-06 |
| Nos2 | 2.858916885 | 1.83E-06 |
| Ccnd1 | -2.378832812 | 1.91E-06 |
| Colq | 2.0669917 | 1.94E-06 |
| Col5a1 | 3.306424306 | 1.97E-06 |
| Tmed6 | 3.808410925 | 1.98E-06 |
| Bmper | 2.129677346 | 2.00E-06 |
| Olfm1 | -2.029862709 | 2.12E-06 |
| Speer2 | 3.190794909 | 2.13E-06 |
| Serping1 | 2.673644756 | 2.15E-06 |
| Derl3 | 3.09138835 | 2.18E-06 |
| Tcstv3 | -2.094803625 | 2.54E-06 |
| Cd79a | 2.207254226 | 2.55E-06 |
| Ogn | 2.142747595 | 2.64E-06 |
| Dcstamp | 2.920262484 | 2.73E-06 |
| Gm6460 | 2.797072445 | 2.78E-06 |
| H2-DMb2 | 2.068478181 | 3.10E-06 |
| Btla | 2.230149559 | 3.15E-06 |
| Rnf183 | -2.197627314 | 3.44E-06 |
| Endou | 2.50067984 | 3.57E-06 |
| Ocstamp | 2.743881107 | 3.78E-06 |
| Tnni2 | -2.080469221 | 4.03E-06 |
| 4933402N22Rik | 2.674608432 | 4.42E-06 |
| Rasal1 | 2.275759791 | 4.60E-06 |
| Cxcl12 | 2.316512628 | 4.68E-06 |
| Speer1 | 2.842771146 | 5.26E-06 |
| Chst3 | 2.395188254 | 5.51E-06 |
| Cacna1s | 2.588399582 | 7.10E-06 |
| Bank1 | 2.215685256 | 1.08E-05 |
| Zcchc18 | 2.327752454 | 1.18E-05 |
| Prg2 | -2.180144047 | 1.43E-05 |
| Gjb2 | 2.044991777 | 1.48E-05 |
| Arg1 | -2.799969255 | 1.52E-05 |
| Mmp23 | 2.030399503 | 1.53E-05 |
| Neurl1b | -2.435198108 | 1.66E-05 |
| Lmnb1 | -2.081533368 | 2.12E-05 |
| Artn | 2.081389872 | 2.22E-05 |
| Gm34484 | 2.041192574 | 2.27E-05 |
| Bend5 | 2.063726341 | 2.34E-05 |
| Hip1r | 2.151205913 | 2.41E-05 |
| Amica1 | -2.005265698 | 2.46E-05 |
| Gm6647 | 2.424130031 | 3.41E-05 |
| Ccl7 | 2.754872247 | 3.60E-05 |
| Ccl2 | 2.189732418 | 9.43E-05 |
| Cpa3 | -2.630753615 | 0.00010072 |
| Ccl24 | -2.536217698 | 0.000105464 |
| Mcpt8 | -2.418199082 | 0.00071594 |
| Prss34 | -2.888934057 | 0.000866845 |

**Table S4. 33 differentially expressed metabolites**

| **Name** | **C number** | **LogFC** |
| --- | --- | --- |
| Riboflavin | C00255 | 1.54705 |
| b-Pseudouridine | C02067 | -2.02698 |
| Uridine | C00299 | -1.87521 |
| Adenosine | C00212 | -1.65205 |
| Biotin | C00120 | -1.05264 |
| N-Acetyl-L-leucine | C02710 | 2.01675 |
| L-(-)-Methionine | C00073 | 1.05205 |
| Methionine sulfoxide | C02989 | 1.11405 |
| Ornithine | C00077 | 2.10546 |
| L-Tyrosine | C00082 | 2.10521 |
| Valine | C00183 | 1.995637 |
| Catechol | C00090 | -1.10549 |
| Hyodeoxycholic Acid | C15517 | -3.11205 |
| Glycocholic acid | C01921 | 2.88705 |
| Stercobilin | C05793 | -3.04158 |
| D-Glucose 6-phosphate | C00092 | -2.54786 |
| Deoxyribose 5-Phosphate | C00673 | -1.40578 |
| 4-Hydroxybenzaldehyde | C00633 | 1.61053 |
| Sildenafil-d3 | C07259 | 2.68704 |
| 2-Hydroxycinnamic acid | C01772 | 2.21405 |
| Skatole | C08313 | 2.88795 |
| DL-Tryptophan | C00525 | 2.49867 |
| Indole-3-lactic acid | C02043 | 1.95765 |
| 7-Methylguanine | C02242 | 1.04956 |
| Guanine | C00242 | 1.66054 |
| Cytosine | C00380 | 1.56654 |
| PI(16:0/16:0) | C00626 | 4.95765 |
| Adenosine monophosphate | C00020 | -3.98056 |
| Phosphatidylinositol-3,4,5-trisphosphate | C05981 | -4.44016 |
| Nitric oxide | C00533 | -4.54935 |
| Phosphatidylinositol-4,5-bisphosphate | C04637 | 5.54046 |
